# Sixty years of linguistic change in the poetry of a Zen practitioner: a longitudinal case study of Gary Snyder

**DOI:** 10.3389/fpsyg.2026.1879502

**Published:** 2026-09-25

**Authors:** Ping Guo, Wei Fan

**Affiliations:** 1 School of Foreign Languages, Henan University of Technology, Zhengzhou, China; 2 Business School, Zhengzhou University, Zhengzhou, China

**Keywords:** computational quantitative content analysis, contemplative transformation, Gary Snyder, longitudinal study, Zen meditation

## Abstract

Can decades of Zen meditation leave measurable traces in the practitioner’s natural language? Long-term contemplative practice is widely understood to reshape emotion regulation, self-referential processing, and perceptual engagement, yet empirical research on these transformations has been fundamentally constrained by two limitations: an observation window of weeks to months at most, and an almost exclusive reliance on self-report and neuroimaging measures that cannot reach the automated, prereflective dimensions of psychological life most directly reshaped by sustained practice. This study applies computational quantitative content analysis to nine major poetry collections by the American Zen practitioner-poet Gary Snyder, spanning six decades (1959-2015), to test three directional hypotheses derived from core Zen concepts: affective settling (*ānxīn*, H1), self-referential attenuation (*anātman*, H2), and perceptual despecification (*ānimitta*, H3). Each hypothesis is held against a rival explanation as a falsification reference. The results reveal career-long changes across three analytical domains: negative-emotion vocabulary declines monotonically while positive and neutral categories show no statistically detectable trend; first-person pronouns, somatic vocabulary, grammatical agency, and cognitive certainty converge in decline across four distinct linguistic layers; embodied sensory specificity thins systematically while vocabulary drifts toward greater abstraction. Changepoint detection offers descriptive corroboration of the periodization. A comparison with a contemporaneous non-practicing poet (Wendell Berry) is descriptive in evidential status: the affective and self-referential trends are significant in Snyder but not in Berry, direct tests of the between-poet differences do not yield differences that are robust across interval-construction methods, and sensory thinning appears in both poets and may reflect processes shared across long poetic careers. These findings constitute what is, to our knowledge, the longest longitudinal linguistic record of a verified long-term Zen practitioner–documenting career-long changes that are consistent with, though not attributable to, sustained practice–and demonstrate the distinctive methodological value of naturally produced creative texts as a comparatively non-reactive observational window for studying long-term contemplative transformation.

## 1 Introduction

Can decades of Zen meditation leave measurable traces in the practitioner’s natural language–and if so, how might we observe them? Empirically testing this question requires satisfying two conditions simultaneously: a longitudinal observation spanning decades, to match the timescale over which deep practice effects are thought to unfold; and a non-intrusive measurement that mitigates reactive biases, capable of capturing automated emotional responses, habitual self-referential narratives, and other deep psychological structures that lie beyond conscious introspection. Cases meeting both conditions are exceedingly rare. The American Zen practitioner-poet Gary Snyder is among the best documented: six decades of sustained, verifiable practice (formal training beginning in Kyoto in 1956) and a longitudinal linguistic archive consisting of nine major poetry collections from *Riprap* (1959) to *This Present Moment* (2015). As creative texts produced without the aim of self-description, poems naturally circumvent demand characteristics and social desirability biases, satisfying the core requirement of non-reactive measurement. At the same time, poetry is a highly edited, aesthetic, and culturally situated form of language; we therefore treat it as a comparatively–rather than a fully–non-reactive window, and (as detailed below) rest our inferences on the less deliberately controlled function-word and syntactic strata rather than on imagery. This study conducts an empirical investigation on that corpus.

Long-term contemplative practice is widely understood to produce enduring changes in emotion regulation, self-referential processing, and perceptual engagement (Davidson et al., 2003; Goleman and Davidson, 2017; Ehmann et al., 2025), yet the empirical study of these transformations is constrained at two fundamental levels. The first is temporal mismatch: laboratory paradigms track practitioners for weeks or months at most, whereas the transformative effects documented in traditional practice contexts are thought to require decades to unfold. A recent review of advanced-meditation research (Ehmann et al., 2025) likewise notes that the evidence base for long-term practitioners remains thin, and that observation windows have not extended far into the decades over which deep practice is thought to unfold. The second constraint concerns the reactivity of the measurement dimension itself: existing research relies almost entirely on self-report questionnaires and neuroimaging paradigms, yet the dimensions most profoundly altered by long-term practice–automated emotional responses, habitual self-referential narratives, prereflective perceptual engagement–are precisely those least accessible through conscious introspection or laboratory tasks (Goleman and Davidson, 2017). What is needed, then, is a comparatively non-reactive observational window: something capable of capturing the practitioner’s psychological structures without requiring description or performance.

Language provides exactly such a window, and poetry offers a form with distinctive methodological virtues: extreme compression gives every lexical choice extraordinary semantic weight; creative production is not aimed at self-description, avoiding demand characteristics; and a series of collections spanning decades yields dense, discrete longitudinal observation points. Pennebaker’s (2011) foundational psycholinguistic research demonstrates that the use of function words–pronouns, emotion vocabulary, cognitive markers–is largely automatized and lies beyond deliberate rhetorical control, making word frequency patterns a reliable index of psychological processes. Crucially, this automaticity is strongest for function-word and syntactic features (pronouns, grammatical subjects, certainty markers), which remain largely outside deliberate control even in carefully edited verse, whereas imagery is more deliberately chosen; we therefore treat the convergence of these less- and more-controlled strata, rather than either alone, as the evidential signal. This resonates with cognitive science accounts of automatized processing (Bargh, 1994; Kahneman, 2011): a large portion of mental life operates outside conscious control and surfaces indirectly through language. Within a neurocognitive poetics framework, Jacobs (2015) further argues that the distributional features of poetic texts can reflect the author’s underlying cognitive-affective schemata independently of rhetorical foregrounding effects. If sustained Zen practice progressively reshapes emotion regulation, self-referential processing, and perceptual patterns, these transformations should emerge over time in the distributional regularities of the practitioner’s language.

Gary Snyder offers an ideal case for testing this proposition. Snyder arrived in Kyoto in 1956 and began formal kōan practice under Miura Isshu Roshi at Rinko-in, a sub-temple of Daitoku-ji, subsequently training under Oda Sesso Roshi–a six-decade record of continuous, documented practice and uninterrupted poetic production (Suiter, 2002; Martin and Snyder, 2014). His nine major poetry collections–from *Riprap* (1959) to *This Present Moment* (2015)–constitute a longitudinal linguistic archive spanning six decades. Crucially, these data are non-reactive: the poems were not composed to describe meditative experience but represent natural creative output, and are therefore free from demand characteristics and social desirability bias.

This study applies computational quantitative content analysis to this corpus, using a longitudinal single-case research design (Flyvbjerg, 2006; Yin, 2018; methodological justification detailed in §4.1). Three directional hypotheses, derived from core Zen concepts, test whether and how six decades of Zen practice leave measurable traces in natural language. The hypotheses correspond to three psychological dimensions–emotion, self, and perception–each accompanied by a rival hypothesis as a falsification reference. H1 (affective settling) predicts that the monotonic decline is specific to negative-emotion vocabulary and does not extend to the positive and neutral categories, consistent with the Zen concept of *ānxīn*, against the rival of affective flattening (predicting an overall decline in emotion vocabulary). H2 (self-referential attenuation) predicts convergent decline across four layers–first-person pronouns, somatic vocabulary, grammatical agency, and certainty assertions–consistent with the doctrine of *anātman*, against the rival of stylistic maturation (predicting stable pronoun frequency with increasing syntactic complexity). H3 (perceptual despecification) predicts systematic thinning of embodied sensory specificity accompanied by a drift toward abstraction, consistent with the concept of *ānimitta*, against the rival of abstract aging (predicting abstraction without corresponding change in the affective and self-referential domains). Career-long change across the three domains is the broader pattern expected under the practice-related interpretation and a principal basis for weighing it against alternative explanations; changepoint evidence is treated separately as descriptive corroboration of the first period boundary (§5.4).

The paper proceeds as follows. Section 2 reviews the relevant literature. Section 3 develops the theoretical framework underlying the three hypotheses. Section 4 describes the corpus, measures, and analytical strategy. Section 5 reports the results. Section 6 discusses the implications and limitations.

## 2 Literature review

### 2.1 The psychology of meditation

A substantial body of empirical research documents the psychological effects of meditation. Davidson et al. (2003), in a landmark study, reported that an eight-week mindfulness programme produced a shift toward left-sided anterior activation in the electroencephalogram–an asymmetry associated with approach-related positive affect–together with a stronger antibody response to influenza vaccination; Hölzel et al. (2011) proposed a four-component model of meditation’s mechanisms of action–attention regulation, body awareness, emotion regulation, and transformation of self-awareness–grounded in neuroplasticity; Tang et al. (2015) synthesized evidence that meditation training enhances attentional network efficiency and reduces emotional reactivity.

On the theoretical taxonomy of practice, Lutz et al. (2008) distinguished two broad families of meditation–focused attention (FA) and open monitoring (OM)–providing a foundation for comparative research. Dahl et al. (2015) extended this into a three-family framework: the attentional family (strengthening attentional regulation and emotional stability), the deconstructive family (dismantling habitual patterns of self-referential processing), and the constructive family (reconstructing perceptual and evaluative orientation). This framework is particularly valuable for understanding long-term integrated practice: deep practitioners typically engage all three families simultaneously, developing across all three dimensions rather than specializing in any one. Laukkonen and Slagter (2021) propose a “from many to (n)one” model, arguing that meditation progressively attenuates the weighting of higher-order predictions, liberating the mind from habitual abstraction–a framework that illuminates the psychological trajectory of long-term practitioners.

These studies, however, span weeks to months at most, falling far short of the cumulative effects documented in decades of traditional practice. Goleman and Davidson’s (2017) systematic review identifies the long-term psychological evolution of “deep practitioners” as an open empirical question; a recent review of research on advanced meditation and long-term practitioners (Ehmann et al., 2025) synthesizes the available evidence and identifies the scarcity of genuinely long-horizon observation as a limiting condition of the field. Van Dam et al. (2018) have offered a systematic critique of the field, pointing to conceptual ambiguity, methodological inconsistency, and insufficient temporal depth. More critically, none of these studies touches the linguistic dimension–the gap this paper addresses.

### 2.2 Psycholinguistics and computational literary studies

Psycholinguistic word frequency analysis provides the methodological foundation for extracting psychological information from language. Pennebaker (2011) demonstrated that word choice–particularly function words–is largely automatized, relatively insulated from deliberate rhetoric and thus able to index psychological processes; Tausczik and Pennebaker (2010) showed that cross-textual comparisons using standardized lexicons effectively predict psychological states, a principle directly applicable to the NRC Emotion Lexicon deployed in this study. Pennebaker and Stone (2003) also reported that vocabulary tends toward abstraction with age, a finding that structures the rival “abstract aging” hypothesis against which H3 is tested.

Several theoretical frameworks from the psychology of literature provide the epistemological grounding for this study. Miall and Kuiken’s (1994) foregrounding theory demonstrates that literary devices induce slowed reading and affective engagement, while “background” linguistic regularities–base-rate word frequencies, function word patterns–furnish indices relatively independent of rhetorical manipulation. Mar and Oatley (2008) characterize literature as “the abstraction and simulation of social experience,” arguing that a text’s linguistic features both reflect and reshape the social-cognitive schemata of author and reader alike. Jacobs (2015), working within a neurocognitive poetics framework, further argues that distributional word frequency patterns in poetry reflect underlying cognitive-affective states without being obscured by rhetorical foregrounding.

Within computational literary studies, word frequency methods have revealed macroscopic patterns in literature: Reagan et al. (2016) used standardized emotion lexicons to identify six basic emotional arc types in fiction; Öhman et al. (2024) extended this approach to 9,000 English novels and found that emotional complexity correlates significantly with literary reception; Underwood’s (2019) concept of “distant evidence” and Rebora’s (2023) critical survey together legitimate frequency-driven methods in literary scholarship. The application of such methods to poetry, however, poses particular challenges. Kim and Klinger’s (2019) central concern is whether word frequency statistics can capture the emotional texture of a form saturated with metaphor and symbolism. Their answer is that such methods analyze distributional patterns rather than deep semantics, and that basic emotion words retain validity even in poetic language (see also De Sisto et al., 2024; Kao and Jurafsky, 2012).

### 2.3 Snyder scholarship

Snyder’s poetry has generated a rich critical tradition, though the analytical frameworks vary substantially across studies, and none examines his work through the lens of deepening practice as a periodizing principle. Murphy (1990, 1992, 2000), the field’s most important scholar, identifies four interwoven threads throughout Snyder’s career: physical labor, Buddhist practice, communion with the natural world, and ecological commitment; his emphasis falls on continuity and simultaneity rather than discrete stages. Gray (2006) situates Zen within the context of cross-Pacific cultural exchange during the Cold War era; Yamazato (1990) organizes Snyder’s creative trajectory around the question of “how to be” and cautions that Snyder drew on multiple Buddhist traditions, not only Rinzai Zen. Hunt (2004) treats the forty-year evolution of *Mountains and Rivers Without End* as the thread running through all periods; Martin and Snyder (2014) foreground meditation as a continuous investment pervading each creative phase.

At the level of intellectual history and linguistic style, related scholarship has likewise remained within qualitative interpretation. Dean (1991) examines Snyder’s relationship to the American cultural unconscious; Barnhill (1997) explores the fusion of “wildness” and the Zen view of nature in his poetry, reading the “Great Earth Sangha” as a vision of community extended beyond the human; Wirth (2017) revisits the ontological dimensions of “mountains and rivers” in Zen poetics against the backdrop of ecological crisis; Bi (2023) reads Snyder through the lens of classical Chinese poetics, noting that his style of “concentrated compression” is deeply inflected by Zen and Daoist aesthetics–yet this observation, too, remains at the level of qualitative description.

### 2.4 The gap at the intersection

Each of these three fields has generated significant findings; yet at their intersection, three substantial gaps remain. First, temporal depth: the psychology of meditation is constrained to observation windows of weeks to months, incapable of capturing the cumulative effects of decades of practice. Second, measurement dimension: existing meditation research relies entirely on self-report and neuroimaging, lacking non-reactive behavioral-level measurement. Third, methodological path: computational literary studies have yet to undertake longitudinal tracking of practice effects in poetry across multiple decades, while the qualitative consensus in Snyder scholarship–that deepening practice accompanies linguistic transformation–has never been subjected to quantitative testing. This study enters that interdisciplinary space, bringing the psychology of meditation, computational literary studies, and Snyder scholarship into dialogue.

## 3 Theoretical framework

The three hypotheses rest on two mutually reinforcing premises. The first: word frequency data capture unconscious, automatized linguistic patterns–function words, basic emotion vocabulary, and grammatical structures are selected without deliberate awareness (Bargh, 1994; Kahneman, 2011; Pennebaker, 2011; Tausczik and Pennebaker, 2010). The second: the psychological structures most directly targeted by Zen practice–emotional reactivity, self-centered narratives, clinging to sensory features–operate at precisely the same structural level, namely the stratum of deep schemata lying beyond deliberate rhetorical control. Together, these premises make natural writing a non-intrusive observational window onto contemplative transformation (Jacobs, 2015; Mar and Oatley, 2008).

Building on these premises, the framework extracts three psychological dimensions from Snyder’s documented practice tradition as the theoretical anchors for the directional hypotheses. The three dimensions map directly onto Dahl et al.’s (2015) three-family classification: emotion regulation (attentional family), deconstruction of self-reference (deconstructive family), and reshaping of perceptual organization (constructive family). The theoretical resources for each dimension are drawn jointly from Zen and Prajñāpāramitā literature, reflecting the multiple intellectual sources of Snyder’s practice rather than presenting a precise account of any single Buddhist tradition; key concepts are defined according to established Buddhist scholarship (Buswell and Lopez, 2014) without making psychological claims about their ontological status. The framework adopts a functional interpretation: what is examined is the observable imprint of these practice dimensions at the level of linguistic behavior (cf. Pargament, 2002, on the distinction between functional explanation and ontological assertion). Snyder’s Zen background provides a documented primary line of practice spanning all three dimensions–formal monastic training in Kyoto from 1956 to 1969 under two teachers, the establishment of the Ring of Bone Zendo at Kitkitdizze after his return to California, and sustained daily zazen and annual sesshins over the following decades–establishing temporal precedence, which is necessary but not sufficient for a causal interpretation.

### 3.1 Ānxīn (affective settling) and H1

*Ānxīn* does not denote the suppression of emotion but the dissolution, through contemplative awareness, of habitual entanglement with emotional states. This mechanism aligns with the “open monitoring” (OM) mode in meditation cognitive science (Lutz et al., 2008): sustained non-judgmental observation of affective states gradually erodes their habitual pull, neither avoiding nor pursuing them. Within Dahl et al.’s (2015) three-family framework, this mechanism corresponds primarily to the emotion-regulation dimension of the attentional family, developing in concert with advances in the deconstructive and constructive families over years of integrated practice. Neuroscientific evidence supports this mechanism: Davidson et al. (2003) reported a shift toward left-sided anterior EEG activation, an established correlate of approach-related positive affect, following an eight-week mindfulness programme; Hölzel et al. (2011) identified emotion regulation as one of four core mechanisms of meditation. Extended to the timescale of decades, the central prediction is not the wholesale exhaustion of emotion vocabulary but the monotonic decline of negative emotion vocabulary–a reduction in clinging to aversive states–while positive and neutral categories do not decline. This pattern is structurally distinguishable from the affective-flattening rival hypothesis, which predicts an overall decline in emotion vocabulary across the board.

H1: As years of practice accumulate, the frequency of negative-emotion vocabulary will decline monotonically, with no significant linear trend in positive or neutral emotion categories.

### 3.2 Anātman (non-self) and H2

*Anātman* is the central doctrine of the Madhyamaka and Zen traditions, holding that the self is not a permanently existing entity but an interdependent assemblage lacking inherent nature; in meditation practice, this understanding moves from conceptual recognition toward embodied realization through sustained contemplative awareness. Within Dahl et al.’s (2015) framework, the actualization of *anātman* corresponds closely to the deconstructive family: the systematic dismantling of habitual cognitive-emotional patterns of self-reference. The psychological expression of anātman is a progressive de-centering–a loosening of the self-centered narrative posture. The modulation of self-referential processing by contemplative practice, and its potential clinical significance, have been discussed in a recent commentary (Sacchet et al., 2024). Pennebaker (2011) demonstrated that first-person pronoun frequency indexes the degree of self-focus; yet the linguistic imprint of “self” extends well beyond pronouns to include somatic vocabulary, grammatical subject choice, and cognitive certainty markers. H2 therefore predicts convergent attenuation across four layers. The stylistic-maturation rival hypothesis predicts stable pronoun frequency with increasing syntactic complexity–a pattern structurally different from multi-level convergent decline.

H2: As practice deepens, the multi-level linguistic markers of self-reference–first-person pronouns, somatic vocabulary, grammatical agency, and certainty assertions–will decline convergently across four layers.

### 3.3 Ānimitta (signlessness) and H3

Ānimitta designates the transcendence of clinging to sensory features (nimitta) as ultimate reality–a core insight of the Prajñāpāramitā tradition, in which the practitioner ceases to grasp at the concrete sensory “marks” of phenomena. Within Dahl et al.’s (2015) framework, this mechanism is associated with the constructive family: practice reconstructs the organization of perceptual experience, moving from grasping at concrete features toward a more open awareness. The predicted linguistic imprint is not the replacement of one sensory modality by another but the systematic thinning of overall sensory specificity–a reduction in the lexical density that anchors poetry in concrete embodied experience–accompanied by a shift toward more abstract, spatially oriented vocabulary with correspondingly longer syllable counts. Pennebaker and Stone (2003) report a general tendency for vocabulary to become more abstract with age, which structures the abstract-aging rival hypothesis. The key distinction between H3 and this rival lies in whether sensory thinning occurs alongside the affective and self-referential trends rather than as abstraction alone. Changepoint analysis is treated separately as a descriptive test of where structural breaks fall relative to the biographical periodization.

H3: As practice deepens, embodied sensory specificity will thin systematically and vocabulary will shift toward greater abstraction and longer syllable counts.

### 3.4 Multidomain change as a discriminating criterion

The three dimensions–emotion regulation, self-reference, and sensory specificity–are theoretically interrelated within Zen soteriology: because there is no self to cling to, emotions lose their habitual “owner” and tend toward settling; as the subject-object duality dissolves, perception is freed from discriminative grasping. This internal coherence generates a key methodological implication: observing change in only one dimension while the other two remain stable would count against a meditation-based explanation and instead point toward some localized stylistic shift. Career-long change across all three domains is the pattern expected under the practice-related interpretation and a principal basis for weighing it against alternative explanations–natural aging, period aesthetic currents, and deliberate artistic development alike. This expectation concerns the direction and temporal development of the indicators; it should not be conflated with the narrower changepoint test, which is reported indicator by indicator in § 5.4.

The three-dimensional framework maps onto Dahl et al.’s (2015) three-family classification in structural correspondence: the ānxīn dimension corresponds to the attentional family’s emotion-regulation modality; the anātman dimension corresponds to the deconstructive family’s systematic dismantling of self-representation; the ānimitta dimension corresponds to the constructive family’s reshaping of perceptual organization. The co-development of all three families in long-term integrated practice is consistent with the pattern of “integrated practice” that Dahl et al. describe. The results reported here are therefore best interpreted in relation to the Dahl-Lutz-Davidson framework–as a possible longitudinal extension of that account to a sixty-year timescale–rather than as a test of predictions the framework specified in advance for this particular pattern of linguistic change. It should also be stressed that the correspondence just drawn is an interpretive mapping. The three Buddhist concepts supply the theoretical rationale for which linguistic indicators to examine and in which direction; they are not themselves measured. What the indicators record is linguistic expression, and the contemplative reading of that expression is an interpretation placed upon it rather than a property established by the measurement.

## 4 Materials and methods

### 4.1 Research design

This study employs a longitudinal single-case research design (Flyvbjerg, 2006; Yin, 2018). Methodologically, the analysis is best characterized as computational quantitative content analysis (Krippendorff, 2018; Grimmer et al., 2022): predefined linguistic categories (emotion, self-reference, perception) are operationalized through validated dictionaries and computational syntactic parsing, then quantified and tested across time. It is distinct from thematic analysis, which inductively interprets latent qualitative themes; here the categories are defined in advance of coding rather than induced from the texts. The quantitative analysis is triangulated with close reading of representative poems, integrating “distant” and “close” reading (Underwood, 2019). Because poetic language is aesthetically crafted and culturally situated, the design deliberately privileges the less deliberately controlled function-word and syntactic strata as primary evidence and treats imagery as corroborative rather than probative. The design is best described as a longitudinal archival single-case study: it is observational throughout, and it differs in kind from single-case experimental research. No intervention was introduced by the investigators; there is no pre-intervention baseline, since Snyder had already been practicing for several years when the earliest analyzed poems were composed; the transitions between phases were neither controlled nor manipulated; and, because practice is cumulative, no withdrawal or reversal of the putative influence is available. The three phases are therefore observational strata within a single continuous life, not experimental conditions, and the evidence they support is correspondingly observational.

Analytical rigor derives from three structural features: (1) each indicator is subjected to three complementary analytical frameworks–which analyze overlapping data from different angles rather than constituting independent replications–with cross-framework consistency serving as the primary evidential standard; (2) PELT changepoint detection is applied to the binned series without presetting the number or timing of breakpoints; (3) alternative hypotheses are explicitly tested in the Discussion. The generalizability of the design follows the logic of analytic generalization–the goal is to generate theoretical propositions testable in subsequent comparative work, not statistical generalization across individuals (Yin, 2018; Gerring, 2007). No multi-person dataset in the meditation science literature comes close to matching the temporal depth and linguistic richness of this corpus; the Snyder corpus constitutes the longest natural language record available for any verified long-term practitioner.

### 4.2 Corpus and practice documentation

#### 4.2.1 Snyder’s practice trajectory

The temporal depth and authenticity of Snyder’s practice are supported by multiple lines of evidence (Suiter, 2002; Gray, 2006; Martin and Snyder, 2014; Snyder, 1999). During the formal monastic period, 1956–1969, Snyder served as attendant to the abbot Miura Isshu at Rinko-in, a sub-temple of Daitoku-ji, and began formal kōan practice there; he subsequently trained under Oda Sesso Roshi–both teacher relationships are well-documented–accumulating approximately 13 years of intensive Rinzai Zen training. After returning to the United States in 1969, he established a meditation community at Kitkitdizze in the Sierra Nevada, integrating zazen into daily work and ecological practice, and maintained daily sitting and annual sesshins over the following decades (Martin and Snyder, 2014). Snyder’s zazen practice predates the analyzed corpus in layered fashion: informal practice is documented from 1949 (tentatively) and from 1952 (regularly)–at Reed College, with Philip Whalen in San Francisco in the early 1950s, and on the Crater Mountain lookout in the summer of 1952, where a daily schedule set aside hours for zazen (Suiter, 2002; Schneider, 2015)–while formal kōan training began in 1956, preceding 89% of the analyzed poems (460 of 517) and both detected changepoints (§5.4). The 57 poems of 1951–1955 were composed during the informal-practice years, before formal training. Temporal precedence, in this layered sense, is necessary but not sufficient for a causal interpretation. English was Snyder’s native language (he was born in San Francisco in 1930); the linguistic patterns analyzed here are therefore not confounded by second-language effects.

#### 4.2.2 Periodization and corpus composition

The study covers Snyder’s nine major poetry collections, grouped into three phases according to the dominant mode of his Buddhist practice. Because the collections appeared in chronological sequence, the phase boundaries coincide with the publication years of the volumes that close the first two groups, 1970 and 1983. Within that frame, however, each poem is assigned to a phase by its actual composition date rather than by the publication date of the volume in which it appeared, following Almon (1979) methodological caution against reading a poet’s development off the publication record; the three collections whose contents straddle a boundary are therefore divided poem by poem (see Supplementary Appendix A). The first boundary marks a substantive change in how practice was conducted. Snyder returned from Japan in 1969 after more than a decade of formal temple training in Kyoto and settled in the Sierra Nevada, where practice moved out of the monastery and into daily work, family life, and ecological activism; this is not merely a change of residence but a change in the form practice took, and Snyder’s biographers describe it in broadly convergent terms (Suiter, 2002; Gray, 2006). The second boundary is of a different kind. It marks the point at which the applied, activist practice of the middle years gives way to the synthesis and settledness of a long-accumulated discipline–*Axe Handles* (1983), organized around the theme of transmission, reads as the completion of that middle phase–but it corresponds to no single documented event and is not datable to a particular year in the way the first boundary is.

T1–Early Practice and Monastic Training Period (through 14 years of formal practice; composition date ≤1970). This period opens with the earliest analyzed poems (1951–1955; 57 poems), composed during Snyder’s years of informal, self-directed zazen before formal training (Suiter, 2002; Schneider, 2015), and spans his 1956 departure for Japan, more than a decade of Kyoto temple practice, and his 1969 return to the United States. His Buddhist engagement traces a trajectory from an intuitive encounter with Buddhist aesthetics (*Riprap* [1959]), through mythic and shamanic explorations of early Buddhism (*Myths & Texts* [1960]), into formal Rinzai Zen training (*The Back Country* [1968]), and toward the integration of practice with marriage and family life (*Regarding Wave* [1970]).

T2–Resettlement and Ecological Practice Period (15–27 years of practice; 1971–1983). After returning to the United States, Snyder settled at the Kitkitdizze community in the Sierra Nevada, established a zendo, and wove Buddhist practice into ecological activism and daily labor. *Turtle Island*(1974) fuses ecological writing, indigenous cultural engagement, and political commitment; *Axe Handles* (1983) turns toward everyday work and intergenerational transmission.

T3–Integration and Maturity Period (40+ years of practice; composition date ≥1984). This period marks deep integration following decades of sustained practice. *Mountains and Rivers Without End* (1996), composed over forty years, achieves a grand synthesis of geology, Buddhist philosophy, and ecological vision (Hunt, 2004). *Danger on Peaks* (2004) embodies the equanimity of a mature practitioner; the title of *This Present Moment* (2015) itself distills a core Zen teaching. This phase is thus defined by the cumulative depth of practice rather than by a dated transition.

Of the nine collections, six fall entirely within a single period; the three collections that span multiple periods are assigned poem by poem according to actual composition date (see Supplementary Appendix A). Eighteen translated poems by Miyazawa Kenji in *The Back Country* and twenty-four Hanshan translations in *Riprap* have been excluded to prevent the act of translation from confounding the linguistic patterns under study. The corpus comprises 517 poems (T1: 236; T2: 145; T3: 136). We should be explicit about what this three-phase division is and is not. It is an operational simplification adopted to give the word-frequency comparisons a tractable grouping variable; it is not offered as a replacement for the existing critical frameworks, which have good reasons to resist periodization altogether. Murphy (1990, 1992, 2000) traces four threads running continuously and simultaneously through Snyder’s career; Yamazato (1990) cautions that Snyder drew on several Buddhist traditions rather than Rinzai Zen alone; Martin and Snyder (2014) present meditation as a continuous investment pervading every creative phase. These finer-grained qualitative readings are a standing reminder that any partition of a sixty-year career sacrifices some of its continuity and complexity. Two features of the present design are intended to offset that cost: Framework A treats career time as a continuous variable and does not use the phase boundaries at all, so the principal trend results do not depend on where the divisions are drawn; and the changepoint analysis (§5.4) locates structural breaks from the data themselves, independently of the qualitative division. Of the two boundaries, the first finds convergent support–it is close both to the documented change in practice setting described above and to the earlier of the two structural breakpoints identified by changepoint detection–while the second does not: the later structural breakpoint falls around 1997, more than a decade after the T2/T3 division, and we therefore do not claim that the changepoint analysis corroborates that division.

### 4.3 Analytical tools and core indicators

The foundational method is word frequency analysis; each dimension employs distinct lexicon sources and analytical strategies.

Affective dimension. The NRC Emotion Lexicon (Mohammad and Turney, 2013)–covering 14,154 English words across ten emotion categories–calculates the frequency of each category per thousand words in each poem. The core indicator is NRC negative-emotion vocabulary frequency, examined alongside positive-emotion vocabulary.

Self-referential dimension. At the lexical level, domain-specific lexicons are constructed following the standard procedure for function word analysis established by Pennebaker (2011): first-person singular pronouns (I/me/my/mine); somatic words (hand/eye/blood/bone, etc.; 40 terms); cognitive vocabulary (certainty words and tentativeness words). At the syntactic level, NLTK is used for sentence tokenization and part-of-speech tagging, with the grammatical subject of each sentence classified into four categories (algorithm details in Supplementary Appendix F).

Perceptual dimension. Domain-specific lexicons cover embodied sensory words (cold/rough/breath/skin/bone, etc.; 56 terms) and sub-categories of nature vocabulary (geological, botanical, faunal, hydrological, atmospheric; 200+ terms). Core indicators are embodied sensory word frequency, five-sense distribution, and mean syllables per word.

Lexicon construction and reliability. Seed terms for the three domain-specific lexicons were drawn from the “Body” and “Perception” categories of LIWC 2022 and extended by the first author through high-frequency words in the Snyder corpus; the second author independently validated the categorization (binary judgment: yes/no). Inter-rater agreement was excellent (Landis and Koch, 1977; Cohen’s κ = 0.92 overall; body lexicon: κ = 1.00, nature lexicon: κ = 0.88, sensory lexicon: κ = 0.88; percent agreement: 100%/97.5%/94.3%); 8 disagreement items were resolved by consensus (category-level details in Supplementary Appendix F, Table F-3). To further assess directional validity, each domain-specific lexicon indicator was compared against the closest available category in the open-source lexical tool Empath (Fast et al., 2016; 200 predefined categories; Supplementary Appendix F, Table F-4): directional agreement was 4/4 across all matched pairs, with collection-level Spearman *r* = 0.53–0.80 (2 of 3 computable pairs reached *p* < 0.05; the fourth pair’s collection-level correlation cannot be computed because Empath registers sadness in only 8.7% of poems, so all nine collection-level medians are zero–see the note to Supplementary Appendix F, Table F-4), confirming the directional validity of the domain-specific lexicons. Together, these procedures–the second-coder validation and the cross-lexicon check above, complemented by close readings anchored in the established scholarly literature on Snyder’s poetry and its Buddhist sources–constitute the linguistic and literary validation of the interpretive layer; the quantitative results themselves are produced by fully reproducible computation and do not depend on any single close reading.

### 4.4 Analytical strategy

#### 4.4.1 Three complementary analytical frameworks

Three complementary analytical frameworks are applied, with cross-framework consistency serving as the evidential adjudication criterion. Framework A (continuous trend, *n* = 517) treats years of practice as a continuous variable, takes each poem as the unit of analysis, and computes Kendall’s τ rank correlation–a measure of monotonic trend that assumes no linear relationship and is robust to outliers. Framework B (collection-level, *n* = 9) aggregates the 517 poems by collection (using medians), orders the nine collections by publication year, and computes Kendall’s τ; its advantage is treating collections as independent observations, addressing the possible within-collection clustering in Framework A. Framework C (period comparison, T1 vs. T3) directly compares T1 (236 poems) and T3 (136 poems) using the Mann-Whitney U test, bypassing the transitional character of T2 and testing the endpoint difference directly.

Evidence adjudication rule: an indicator reaching statistical significance in all three frameworks is recorded as “3/3” and treated as robust evidence; significance in two frameworks is recorded as “2/3” and treated as moderate evidence.

#### 4.4.2 Multiple comparisons correction and full indicator disclosure

The study defined for the present analysis 15 measures corresponding to the three hypotheses (H1: NRC negative composite and five sub-categories; H2: pronouns, somatic words, nature-to-self subject ratio, certainty/tentativeness ratio; H3: embodied sensory words, tactile words, sensory evenness, atmospheric words, mean syllables per word), plus 8 exploratory derived indicators (e.g., somatic-to-nature word ratio, body-to-nature word ratio). All indicators were subjected to Benjamini-Hochberg FDR correction based on raw *p*-values for all 23 indicators; results with corrected *p* < 0.05 are reported in Table 1. The complete list of tested indicators appears in Supplementary Appendix E, Table E-1. These measures were defined in the course of the present analysis rather than registered in advance; all of them are reported, none was dropped after inspection, and the FDR correction is applied across the full set, so that the disclosure is complete.

**TABLE 1:** Indicators reaching significance in all three analytical frameworks.

No.	Indicator	Dimension	τ (*n* = 517)	Bootstrap 95% CI	Direction	Frameworks
1	Mean syllables per word	*Ānimitta*	+0.188***	[+0.059, +0.260]	↑	3/3
2	NRC negative affect (composite)	*Ānxīn*	−0.169***	[−0.240, −0.066]	↓	3/3
3	Somatic word frequency	*Anātman*	−0.158***	[−0.175, −0.052]	↓	3/3
4	Sensory evenness	*Ānimitta*	−0.119***	[−0.176, −0.036]	↓	3/3
5	Nature-to-self subject ratio	*Anātman*	−0.130***	[−0.215, −0.049]	↓	3/3
6	Tactile word frequency	*Ānimitta*	−0.129***	[−0.178, −0.050]	↓	3/3
7	Somatic-to-nature word ratio	*Anātman*	−0.127***	[−0.141, −0.036]	↓	3/3
8	Embodied sensory word frequency	*Ānimitta*	−0.115***	[−0.184, −0.027]	↓	3/3
9	NRC sadness	*Ānxīn*	−0.101**	[−0.155, −0.017]	↓	3/3
10	Atmospheric word frequency	*Ānimitta*	−0.100**	[−0.168, −0.011]	↓	3/3
11	I/me/my pronoun frequency	*Anātman*	−0.086**	[−0.150, +0.011]†	↓	3/3
12	Total sensory word frequency	*Ānimitta*	−0.077*	[−0.140, +0.021]†	↓	3/3

Dimension labels are interpretive mappings, naming the hypothesis each indicator serves rather than a direct measurement of the Buddhist concept: Ānxīn = affective settling; Anātman = self-effacement; Ānimitta = perceptual transformation. All indicators reach *p* < 0.05 across Frameworks A (continuous trend, *n* = 517), B (collection-level, *n* = 9), and C (T1 vs. T3 comparison). Bootstrap 95% CIs are based on 1,000 collection-blocked resamples, percentile method (complete CIs in Supplementary Appendix F, Table F-5). †CI spans zero, reflecting the greater uncertainty of these weaker effect-size estimates under collection-blocked resampling; point-estimate direction is consistent across all three frameworks, and these indicators are treated as “moderate evidence.”

*p < 0.05, **p < 0.01, ***p < 0.001 (Framework A values shown).

#### 4.4.3 Time-series robustness

Framework A uses each poem as a longitudinal data unit; adjacent observations may exhibit positive autocorrelation, potentially making standard Kendall’s τ significance tests overly optimistic. Two robustness checks address this: (1) block bootstrap based on collection-level blocks (1,000 resamples), reporting bootstrap 95% CIs (Table 1 note; complete CIs in Supplementary Appendix F, Table F-5); (2) residual autocorrelation function analysis (Supplementary Appendix F, Figure F-1) shows that autocorrelation decays to negligible levels at lag > 1 for all core indicators. Of the 12 core indicators, 10 have block bootstrap CIs that exclude zero; the remaining 2 (I/me/my pronoun frequency and total sensory word frequency) have CIs that span zero, reflecting greater uncertainty in these weaker effects under collection-blocked resampling (details in Supplementary Appendix F, Table F-5). Time-series dependence does not materially alter the core conclusions.

#### 4.4.4 Changepoint detection and inferential scope

Analyses are supplemented by PELT changepoint detection (Killick et al., 2012). The procedure is applied not to the poem-level series but to a thirteen-point aggregate series obtained by binning the 517 poems into five-year windows by composition year and taking the mean of each window; the cost function is L2 with a minimum segment length of two time points. The number and timing of breakpoints are not preset but determined by the penalty parameter, and stability was checked across penalties from 1 to 20 (algorithm details in Supplementary Appendix F). To weigh this observational interpretation, we consider several credibility considerations rather than criteria for causal identification: (1) temporality: informal zazen practice is documented from 1949/1952 and formal training from 1956 (§4.2), so practice in some form precedes effectively the entire corpus, and formal training precedes 89% of the analyzed poems and both detected changepoints–temporal precedence in this layered sense is necessary but not sufficient for a causal interpretation; (2) biological plausibility: the direction of change aligns with the mechanistic predictions of meditation cognitive science; (3) coherence: the earlier of the two detected changepoints falls close to the first of the qualitatively defined period boundaries, providing partial descriptive corroboration of the periodization (treated as corroborative, not confirmatory; the later changepoint does not align with the second boundary, as reported in §5.4); (4) consistency: convergence across the three complementary frameworks, which approach overlapping data from different angles, reduces the probability that the pattern is an artifact of any single analytical choice. We do not treat years of practice as an independent dose variable: in this single-case design, practice years are near-collinear with chronological age, historical time, and career stage, so dose-response reasoning does not apply. Nevertheless, this study should be understood as longitudinal observational evidence rather than causal proof.

### 4.5 Comparison case: Wendell Berry

A single-subject design cannot, on its own, separate the effects of practice from those of chronological aging, because in Snyder the two advance in lockstep. To probe this collinearity, we applied the same indicators and statistical procedures to a purposively selected comparison poet, and the choice of that poet is central to the design’s logic. We selected Wendell Berry (b. 1934) in the spirit of a most-similar comparative case: the two cases are matched as closely as the archives allow on plausibly confounding attributes, so that the comparison can serve as a descriptive probe with an asymmetric evidential function: where a trend appears in both poets, it cannot be regarded as distinctive to Snyder’s case; where it appears in Snyder alone, the sample pattern is reported descriptively and, absent an established between-poet difference, is not used to narrow the space of alternative explanations. Berry approximates this ideal unusually well. He belongs to the same literary generation as Snyder; he writes across a comparably long career (his New Collected Poems (Berry, 2012), covering 1964–2010, comprises 280 poems in eleven constituent volumes); and, most importantly, he works in the same nature/agrarian thematic register that might otherwise be thought to drive Snyder’s trajectory toward calm, abstraction, and a receding self. Yet Berry is a lifelong Christian rather than a Zen practitioner. The two poets are thus closely matched on the two confounds most at issue–aging and nature-poetry theme–while differing in the factor under study. This matching makes the comparison a deliberately conservative, hard probe: because Berry resembles Snyder on exactly the attributes most likely to produce a parallel late-career drift, he is, if anything, among the poets most likely to reproduce Snyder’s pattern, so that a parallel trend observed against so similar a case bears directly on whether the change can be regarded as distinctive to Snyder; a divergent sample pattern, by contrast, is reported descriptively and does not by itself adjudicate among explanations. The design further carries a directional implication–if Snyder’s affective and self-referential changes were artifacts of aging or genre alone, broadly parallel trends in a generationally and thematically matched non-practitioner would be the compatible pattern–so the analysis functions asymmetrically: parallel trends in the comparison poet count against treating a change as distinctive to Snyder, whereas divergent sample patterns, in the absence of an established between-poet difference, are reported descriptively and carry no inferential weight against the alternatives. Following the procedure used for Snyder, each Berry poem was assigned to an early, middle, or late period by the publication year of its constituent volume, with career time (years since the poet’s first collection) as the continuous predictor. Two asymmetries of the comparison should be stated plainly. First, the temporal index differs in resolution: Snyder’s poems are positioned by documented poem-level composition years, whereas no comparably documented poem-level composition dates exist for Berry, so the Berry analysis uses the publication year of the constituent volume–eleven time points, with all poems within a volume tied. New Collected Poems excludes Berry’s separately published, year-dated Sabbath sequences, so the supplementary metadata contain no independently sourced poem-level composition date for the analyzed corpus; the few poems whose titles bear explicit dates are far too few to support a corpus-wide index, and assigning composition years to a small minority while the remainder stayed volume-dated would itself compromise comparability. The Berry trend estimates are accordingly coarser and less precise, and the two poets’ coefficients are not directly comparable on equal footing; in the supplementary metadata the Berry composition-year field carries this publication-year proxy and is flagged as such (year_source = pub_year_proxy). Second, the Berry corpus is an author-curated retrospective selection rather than a complete reproduction of the constituent volumes: New Collected Poems reprints the roughly two hundred poems Berry himself selected from his first eight collections for Collected Poems 1957–1982, together with the poems of Entries, Given, and Leavings. Period-differential self-selection by the poet cannot be excluded, and this is a limitation of the longitudinal comparison. Finally, to test the between-poet contrast directly rather than by comparing patterns of significance, we estimate the difference in poem-level Kendall’s τ, τ(Snyder) −τ(Berry), for each Table 2 indicator, with percentile 95% confidence intervals from a collection-blocked bootstrap (*B* = 20,000; collections resampled with replacement independently within each poet). The percentile construction is pre-specified as the primary analysis for consistency with the Table 1 intervals; basic and bias-corrected and accelerated (BCa) intervals are reported as sensitivity analyses in Supplementary Appendix G. The estimand is the poem-weighted difference in Kendall’s τ between the two corpora as assembled; collections, rather than individual poems, serve as the resampling units.

**TABLE 2:** Snyder vs. Berry – career-trend Kendall τ and between-poet difference tests for core indicators.

Indicator	Dimension	Snyder τ	Berry τ	Δτ (S−B) [95% CI]	Interpretation
NRC negative affect	Affect (H1)	−0.169***	−0.055 n.s.	−0.113 [−0.212, +0.006]	Practice-consistent (descriptive)
First-person pronouns (I/me/my)	Self (H2)	−0.086**	+0.001 n.s.	−0.087 [−0.245, +0.049]	Practice-consistent (descriptive)
Certainty/tentativeness ratio	Self (H2)	−0.085**	−0.065 n.s.	−0.020 [−0.165, +0.184]	Practice-consistent (descriptive)
Mean syllables per word	Perception (H3)	+0.188***	+0.054 n.s.	+0.134 [−0.007, +0.251]	Practice-consistent (descriptive)
Somatic (body) words	Self/Perception	−0.158***	−0.210***	+0.052 [−0.076, +0.228]	Shared (descriptive)
Atmospheric words	Perception (H3)	−0.100**	−0.205***	+0.105 [−0.047, +0.250]	Shared (descriptive)
Total sensory words	Perception (H3)	−0.077*	−0.170***	+0.093 [−0.078, +0.258]	Shared (descriptive)
Tactile words	Perception (H3)	−0.129***	−0.121**	−0.008 [−0.136, +0.128]	Shared (descriptive)
Nature-to-self subject ratio	Self (H2)	−0.130***	−0.180***	+0.049 [−0.119, +0.193]	Shared (descriptive)

Kendall τ against career time (Snyder: practice years; Berry: years since first collection). “Practice-consistent (descriptive)” labels a within-poet pattern–significant trend in Snyder, none in Berry–and carries no inferential claim about the between-poet difference; “Shared (descriptive)” labels significant within-poet trends in both poets. Δτ = τ(Snyder) −τ(Berry), with percentile 95% CIs from a collection-blocked bootstrap (*B* = 20,000; collections resampled with replacement independently within each poet), pre-specified as the primary analysis for consistency with Table 1; sensitivity intervals (basic and BCa) appear in Supplementary Appendix G. All percentile CIs include zero: no between-poet contrast is statistically established under the available temporal indices, and no inferential conclusion rests on the between-poet distinction. For negative-emotion frequency and mean syllables per word the sensitivity intervals reported in Supplementary Appendix G differ from the primary intervals in whether zero is excluded, so those two contrasts are sensitive to interval construction and are treated as suggestive only; for the certainty-to-tentativeness ratio, whose coefficients are close (−0.085 vs. −0.065), no difference is indicated under any construction.

*p < 0.05, **p < 0.01, ***p < 0.001.

## 5 Results

### 5.1 Affective settling: the monotonic decline of negative-emotion vocabulary

The contrast between the emotional texture of the early and middle collections is striking. In *Myths & Texts* (1960), “Logging 14” reads: “The groves are down/cut down… Trees down/Creeks choked, trout killed, roads”–the ancient groves felled, streams choked, trout killed. The negative affect is undisguised; the violence of ecological destruction reverberates in every verb. By *Turtle Island* (1974), “Covers the Ground” confronts comparable ecological transformation in California through a strikingly different linguistic strategy: the poem catalogs modern ground-coverings in a cascade of nouns–“concrete freeway, overpass, underpass/exit floreals, entrance curtsies, railroad bridge”–and closes with the near-neutral statement “us and our stuff just covering the ground.” Not a single negative-emotion word appears; the cut/choked/killed violence is gone. Ecological degradation reaches the reader through objective enumeration rather than affective labeling–and this pattern can be read as consistent with an ānxīn-related interpretation: not the avoidance of feeling, but the waning need to organize perception through affect-laden vocabulary.

NRC Lexicon analysis quantitatively confirms this observation: negative-emotion vocabulary frequency declines monotonically from T1 to T3, corroborated by all three complementary analytical frameworks (τ = −0.169, *p* < 0.001; see Figure 1; period means and full test results in Supplementary Appendix B, Tables B-1, B-2). The differentiation across sub-categories carries crucial theoretical weight: all five negative-emotion types–anger, fear, sadness, disgust, and surprise–decline continuously from T1 to T3, with disgust and sadness showing the steepest decreases. By contrast, none of the four positive or neutral categories–positive (composite), trust, joy, or anticipation–exhibits a significant linear trend. This pattern–a monotonic decline in negative-emotion vocabulary with no detectable trend in the positive and neutral categories–is consistent with affective settling (ānxīn) and tells against the affective-flattening rival hypothesis, which predicts an overall decline in emotion vocabulary–a prediction the data do not support. The pattern approaches the classical Zen understanding of *ānxīn*: emotions settle naturally through awareness, neither suppressed nor pursued.

**FIGURE 1:**
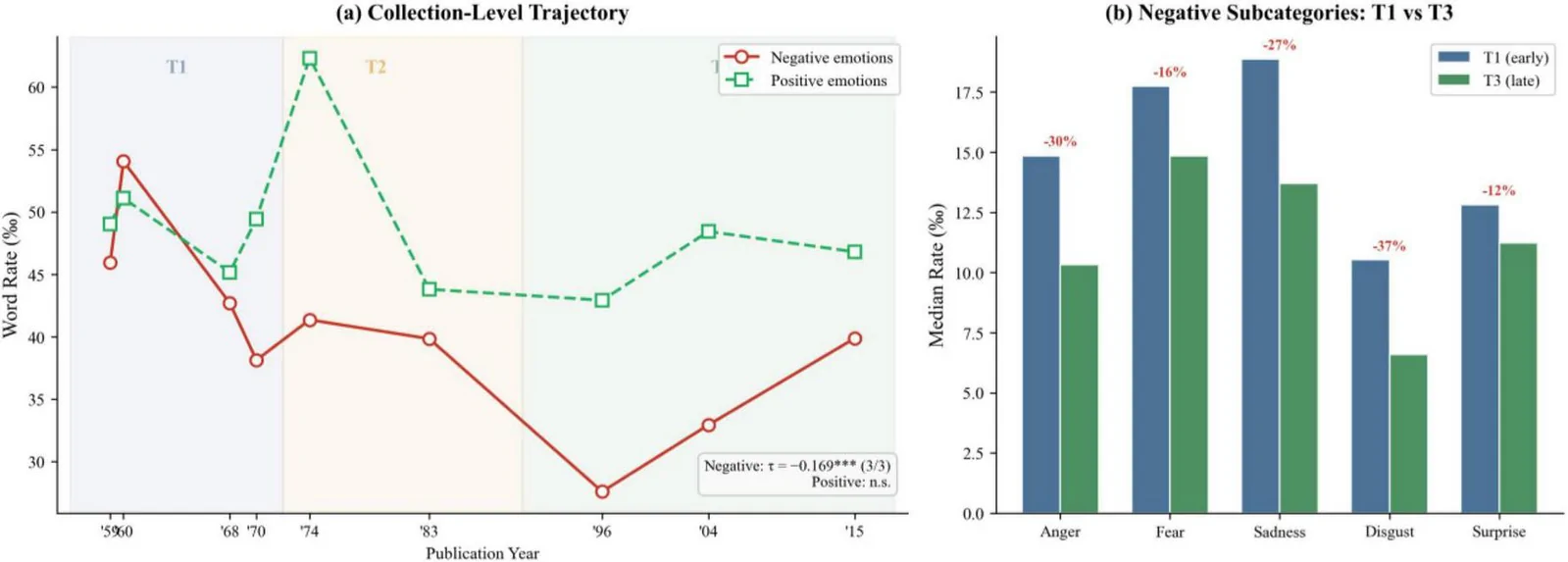
Collection-level trajectory of NRC negative-emotion word frequency. **(a)** Collection-level mean negative- and positive-emotion word rates across the nine collections; **(b)** median rates of five negative-emotion subcategories in T1 versus T3, with percentage changes annotated. ***p < 0.001; n.s., not statistically significant. The coefficient shown is the Framework A Kendall tau; (3/3) denotes significance in all three analytical frameworks.

It is worth noting that the decline in negative-emotion vocabulary does not signal a withdrawal of engagement. “Mother Earth: Her Whales,” read by Snyder at the 1972 United Nations Conference on the Human Environment–“How can the head-heavy power-hungry politic scientist/…bureaucrats/Speak for the green of the leaf?”–contains virtually no standard negative-emotion vocabulary; its condemnatory force comes from the accumulation of rhetorical structure and questions. This observation further supports H1: the observed shift is consistent with changes in affective expression associated with long-term contemplative practice, reshaping the expressive texture of the poems, without weakening their critical engagement.

### 5.2 The retreat of “I”: multi-level convergent evidence for self-effacement

In the early work, “I” is an omnipresent force. This is nowhere more vivid than in the love poems: in *The Back Country*, “Four Poems for Robin” (1965) reads: “I saw you/One time. You were strange./And I was obsessed with a plan”; “I’ve always known/where you were”; “I thought I must make it alone. I/Have done that.” A single poem accumulates first-person pronouns at high density, with the speaker’s ego occupying every syntactic position; love is organized as the narrative of tension between “I” and “you.”

By “Cross-Legg’d” (1984, collected in *Mountains and Rivers Without End*), an intimate moment with a lover is rendered through a fundamentally different linguistic strategy: “Cross-legg’d under the low tent roof,/dim light, dinner done,/drinking tea…/Love made, poems, makyngs,/always new, same stuff/life after life,/as though Milarepa/four times built a tower of stone/like each time was the first.” The poem is virtually devoid of explicit grammatical subjects; actions present themselves; the boundary between “I” and “you” dissolves in the ellipsis. The closing allusion to Milarepa reframes love–no longer the ego’s obsessive pursuit, but the practitioner’s meeting in the present moment. From “I was obsessed” to the subjectless “dim light, dinner done, drinking tea”–the retreat of self-reference reads clearly within a single thematic domain. This pattern is not well captured by the stylistic-maturation rival considered here, which predicts stable pronoun frequency with increasing syntactic complexity, although other forms of literary and biographical development remain possible; the data show instead that pronoun frequency declines continuously alongside an increase in syntactic ellipsis.

Unlike the affective dimension, where a single indicator suffices, self-effacement manifests across multiple linguistic strata. This study traces the transformation through four independent analytical layers, whose results converge into a coherent picture (complete statistics in Supplementary Appendix C).

Layer 1: Lexical frequency. The frequency of first-person singular pronouns (I/me/my) declines significantly from the early to the late period, confirmed across all three frameworks (τ = −0.086, *p* = 0.007; Supplementary Appendix C, Table C-1).

Layer 2: Somatic language. Somatic word frequency (hand/eye/blood/bone, etc.) drops sharply from early to late period (τ = −0.158, *p* < 0.001); the ratio of somatic to nature words also declines continuously, reaching significance in all three frameworks. Somatic reference–the most immediate material vehicle of the self in language–thins markedly across the career; as the Berry comparison shows (§5.5, Table 2), a comparable and indeed steeper thinning appears in the non-practicing poet, so this layer is read as a shared feature of long poetic careers rather than as practice-specific evidence.

Layer 3: Syntactic structure. Syntactic subject analysis tokenizes each poem into sentences and part-of-speech tags them (NLTK), classifying the subject of each sentence by identifying the first pronoun or noun in the sentence-initial region (algorithm details in Supplementary Appendix F). The nature-to-self subject ratio–the count of nature subjects divided by the count of self subjects–declines continuously, from a T1 mean of 0.61 to 0.11 in *This Present Moment* (2015), reaching significance across all three frameworks (τ = −0.130, *p* < .001; Supplementary Appendix C, Table C-3). This decline reflects a thinning of nature subjects rather than a retreat of self subjects, whose rate shows no reliable trend across the three phases (Supplementary Appendix C, Table C-3); we therefore report it as a change in the composition of sentence subjects, not as direct evidence of self-retreat. Qualitatively, the late poems do tend toward the omission of explicit agents of all kinds, with actions and phenomena increasingly presented as arising of themselves, but this observation rests on close reading rather than on the ratio.

Layer 4: Cognitive stance. Certainty words (always/never/definitely/certainly) decline from early to late period, while tentativeness words (maybe/perhaps/might/somehow) increase significantly; the ratio of certainty to tentativeness words declines continuously (τ = −0.085, *p* = 0.008; Supplementary Appendix C, Table C-4). The early poems are replete with always/never-style assertions; the late work moves toward a more open cognitive posture.

These four layers–pronoun frequency, somatic vocabulary, syntactic subject, and cognitive vocabulary–track the linguistic imprint of “self” from distinct angles, and all four decline. They do not, however, decline for the same reason: the pronoun and certainty trends bear directly on self-reference, whereas the somatic decline is shared with the comparison poet (§5.5) and the subject ratio falls because nature subjects recede. Taken together they support a qualified finding of self-effacement, carried chiefly by the pronoun and certainty evidence (see Figure 2). These two carrying indicators themselves differ in robustness status–first-person pronouns meet all three analytical frameworks though with a collection-blocked bootstrap CI spanning zero, whereas the certainty-to-tentativeness ratio meets Frameworks A and C but not B (Table 1 note; Supplementary Appendix C, Table C-4; §6.1)–and the H2 reading weighs them accordingly.

**FIGURE 2:**
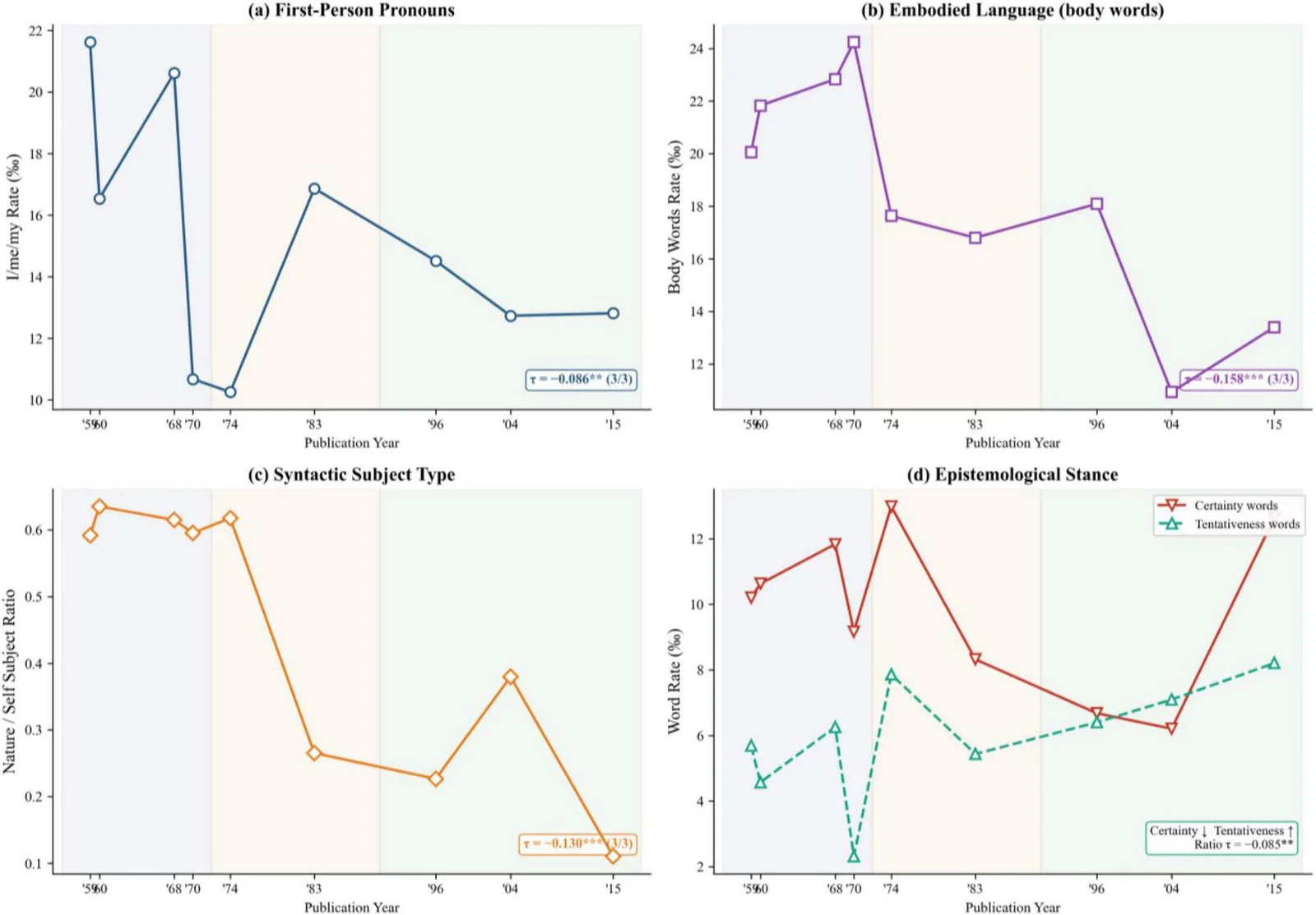
Four linguistic layers bearing on self-reference. **(a)** First-person pronoun frequency; **(b)** somatic word frequency; **(c)** nature-to-self subject ratio; **(d)** certainty and tentativeness word frequencies; the coefficient in panel (d) refers to the certainty-to-tentativeness ratio. *p < 0.05, **p < 0.01, ***p < 0.001.

### 5.3 The transformation of perception: from specificity to signlessness

The transformation in the perceptual dimension is the most directly visible. The T1 high-frequency content words (TF-IDF top 20, function words excluded) cluster around concrete sensory and material experience: cold ⋅ stone ⋅ men ⋅ smoke ⋅ wild ⋅ hair ⋅ hot ⋅ hills–cold and heat, skin and bone, smoke and fire. The poet dwells within the five aggregates, encountering the world through material form (*rūpa*) and sensation (*vedanā*). In “3 days heat, after 5 days rain” (1953) from Riprap: “Down valley a smoke haze/3 days heat, after 5 days rain/Pitch glows on the fir-cones/Across rocks and meadows/Swarms of new flies”–in five lines the poem engages sight (smoke haze, glowing pitch), temperature (3 days of heat), touch (the dampness after rain), and hearing (the swarming of flies). Sensory features anchor the poet’s relationship with the material world at high density.

T2 exhibits transitional characteristics. *Turtle Island* marks the peak of five-sense word density; the collection’s intense ecological focus drives visual, auditory, and tactile vocabulary to their highest levels. In “Front Lines”: “The edge of the cancer/Swells against the hill–we feel/a foul breeze”; “A chainsaw growls in the gorge”; “The skinned-up bodies of still-live bushes”–smell (a foul breeze), hearing (the chainsaw’s growl), touch (swelling, skinning), and sight are all mobilized in the service of ecological critique’s rhetorical intensity.

The pivotal shift in T3 is not the replacement of one category of imagery by another but the thinning of “features” in the language–a systematic dilution of the entire sensory dimension. The T3 high-frequency words point toward space, orientation, and movement: valley ⋅ ridge ⋅ north ⋅ south ⋅ walk ⋅ time ⋅ forest ⋅ place. Vocabulary shifts from sensorially rich words (T1: cold/stone/hot) to spatial words that carry no concrete sensory attribute (T3: valley/ridge/miles)–see Figure 3. Quantitative analysis confirms the systematicity of this shift (complete results in Supplementary Appendix D). Embodied sensory word frequency follows an inverted-U trajectory: sensory density increases slightly in T2–possibly reflecting the period’s concentrated ecological themes–with systematic thinning emerging only in T3; across the full career the continuous decline is significant (Kendall’s τ = −0.115, *p* < 0.001), with the T1 versus T3 endpoint contrast reported separately under Framework C (Supplementary Appendix C, Table C-1). Five-sense analysis reveals further detail: tactile words (touch/cold/warm/rough/smooth, etc.) show the steepest decline, while visual and auditory vocabulary roughly returns to T1 levels after the T2 peak. The sense retreating first is the most intimate–touch–while the most distal–sight–shows no statistically detectable decline. Sensory evenness–an index of how evenly sensory reference is distributed across the five modalities, computed as one minus the share of the dominant modality (Table 1)–also declines significantly, indicating that the T3 sensory thinning is not a redistribution from vision toward other modalities but a wholesale de-sensorization. What the measurements establish is a change in the distribution of sensory vocabulary; reading that change as an expression of ānimitta is an interpretation offered here, not a finding the indicators themselves deliver.

**FIGURE 3:**
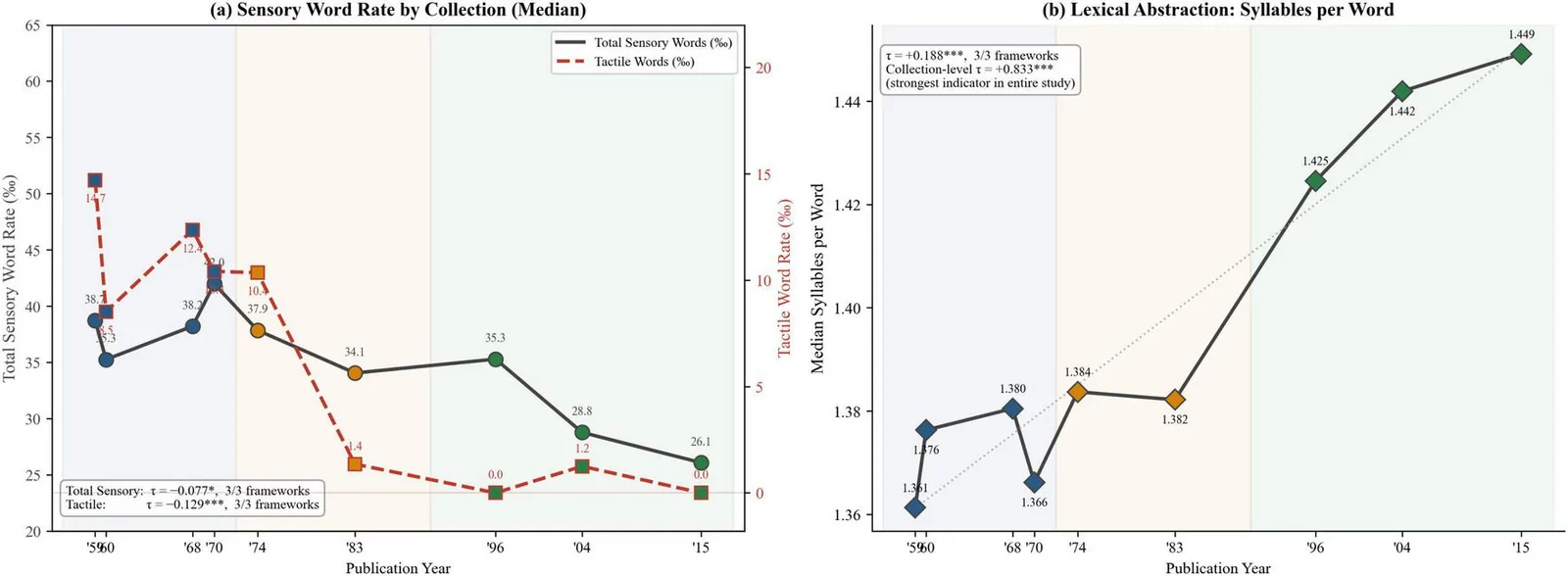
Perceptual transformation – sensory retreat and lexical abstraction. **(a)** Collection-level median total sensory word rate (left axis) and tactile word rate (right axis), per 1,000 words; **(b)** collection-level median syllables per word. Horizontal axes show collection publication years. *p < 0.05, **p < 0.01, ***p < 0.001.

Paralleling the sensory retreat is a morphological change in the vocabulary itself. Mean syllables per word rises steadily from early to late work, yielding the largest effect size in the study (τ = +0.188, *p* < 0.001; Supplementary Appendix D, Table D-3). T1’s core vocabulary is overwhelmingly monosyllabic (stone/cold/dark/men/snow); T3 sees a significant increase in polysyllabic, more abstract words (mountain/valley/ancient/distance/silence). This pattern partially overlaps with Pennebaker and Stone (2003) abstract-aging hypothesis. In the descriptive Berry comparison (§5.5), the rise in lexical abstraction has no significant counterpart trend in the matched non-practicing poet–though the between-poet difference itself is not established–even though parallel sensory thinning is also observed in Berry and may reflect processes shared across long poetic careers.

### 5.4 Changepoints and multidomain change

Are the changes in these three dimensions gradual, or do they exhibit critical turning points? Applied to the five-year-binned series (thirteen time points; L2 cost, minimum segment length of two), PELT identifies two structural breakpoints without their number being preset. At pen = 10–the value used for the bootstrap analysis–three of the five core indicators–negative-emotion frequency, first-person pronoun frequency, and somatic word frequency–yield breakpoints, and in each case they fall in the same two intervals–the 1970–1975 and 1995–2000 intervals–which we report as approximately 1972 and approximately 1997; the remaining two indicators, mean syllables per word and the nature-to-self subject ratio, yield no breakpoint at any tested penalty (see Figure 4 and Supplementary Appendix F, Table F-1). Sensitivity is not identical across penalties: the pronoun and somatic series retain both locations from pen = 1 through pen = 20, whereas at pen = 20 the negative-emotion series retains only the earlier location. Two sources of uncertainty attach to these locations. The five-year binning gives them an inherent resolution of about ±2.5 years, which is why they are reported as approximate. Beyond that, the bootstrap analysis reported in Supplementary Appendix F (Supplementary Appendix F, Table F-1b) shows the earlier breakpoint to be the more stable of the two, its modal location recovered in 87–99 per cent of resamples across the three indicators, against 76–93 per cent for the later one, whose confidence interval spans the 1995–2000 range. The approximately 1972 breakpoint corresponds to Snyder’s departure from the Kyoto temples and his resettlement in California, where he subsequently established the Kitkitdizze meditation community, integrating Zen practice with daily labor and ecological activism. The two breakpoints stand in different evidential positions. The earlier one falls close to an independently documented change in the setting and form of practice, and to the T1/T2 boundary. The later one has no such counterpart: it falls more than a decade after the T2/T3 boundary, and no independently documented transition in Snyder’s practice corresponds to it. We therefore do not offer a practice-based interpretation of the later breakpoint, and we are careful to distinguish transitions in poetic production–the appearance of a major collection, for instance–from transitions in practice itself, which the former does not establish. Taken together, the changepoint evidence corroborates the first period boundary and not the second; we treat it as suggestive rather than as decisive evidence against an aging account, for which the cross-poet comparison (§6.2) provides a targeted probe.

**FIGURE 4:**
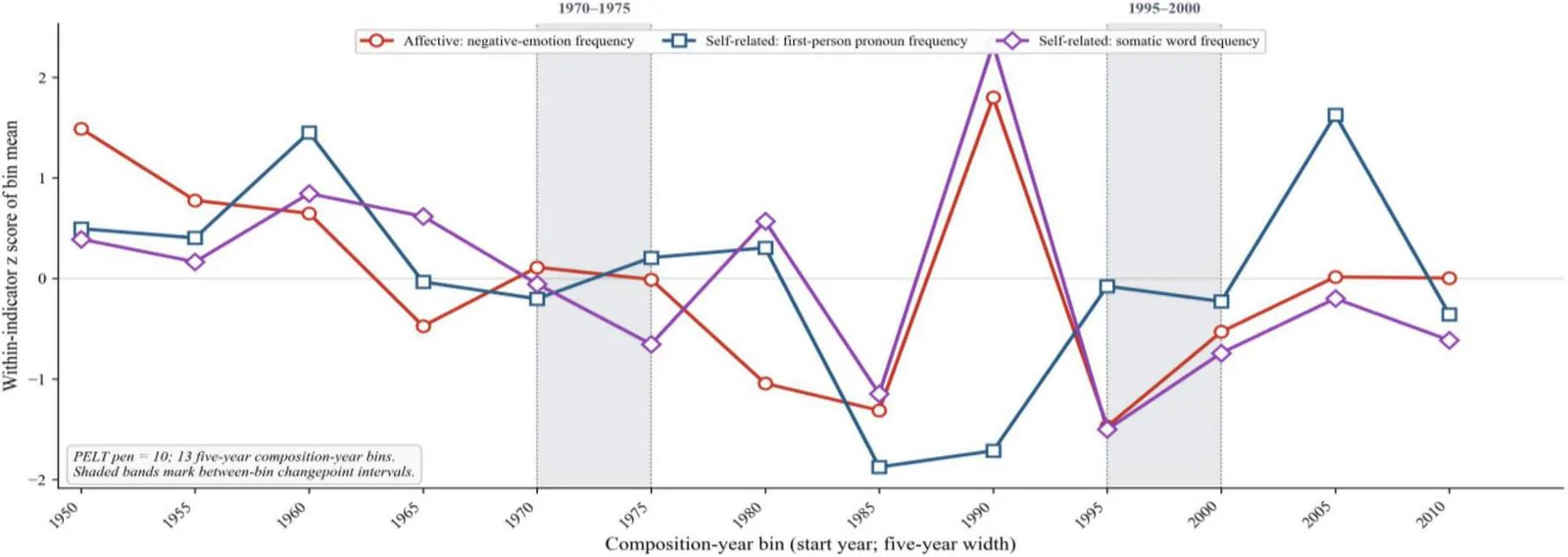
Five-year composition-year-bin trajectories of the three indicators with changepoints at pen = 10. Points are means for the 13 bins and each series is standardized within indicator; shaded bands mark the 1970–1975 and 1995–2000 intervals. The series comprise one affective indicator (negative-emotion frequency) and two self-related indicators (first-person pronoun and somatic word frequencies).

Across the full career, significant continuous trends appear in all three theoretical domains–affective settling, self-referential attenuation, and perceptual transformation–so the multidomain result is not limited to an isolated measure. The changepoint result is narrower: at pen = 10, coincident locations occur in one affective indicator and two self-related indicators; the H3 indicator included in the five-series PELT check (mean syllables per word) yields no breakpoint. These two forms of evidence should therefore be kept distinct. Negative-emotion vocabulary, first-person pronouns, somatic and sensory vocabulary, and certainty assertions all decline continuously. Table 1 summarizes the 12 indicators reaching statistical significance in all three complementary analytical frameworks.

Eight of these 12 indicators are also reported in Table 2. Among them, negative-emotion frequency, first-person pronouns, and mean syllables per word carry the defined label “Practice-consistent (descriptive)”–a significant within-poet trend in Snyder and none in Berry, with the primary percentile tests establishing no reliable between-poet difference (§5.5)–whereas the somatic, sensory, atmospheric, and subject-ratio indicators also show significant within-poet trends in the non-practicing comparison poet and are labeled “Shared (descriptive),” so that they cannot be regarded as uniquely practice-related. The certainty-to-tentativeness ratio is additionally reported in Table 2 but is not included in Table 1. The nature-to-self subject ratio is computed as the count of nature subjects divided by the count of self subjects; its decline reflects a reduction in nature subjects rather than in self subjects, whose rate shows no reliable trend across the three phases, and Table 2 shows the same decline in the comparison poet. Table 1 includes only indicators significant in all three frameworks (3/3); indicators not meeting this criterion are disclosed in Supplementary Appendix C, Table C-4 (three-framework results) and Supplementary Appendix F, Table F-2 (FDR-corrected full list). The certainty-to-tentativeness ratio (τ = −0.085) meets Frameworks A and C but not B, and is therefore used in §5.2, Table 2, and §6.1 with its robustness status stated there.

Taken together, these career-long trends span multiple measures, but the observational design does not identify a common underlying cause. Their breadth is consistent with the integrated-practice account (Dahl et al., 2015), in which practice is associated with change across multiple dimensions rather than a single one. The convergence of the affective and self-referential dimensions across indicators and across the three analytical frameworks within the Snyder corpus is a principal basis for weighing the meditation hypothesis against alternative explanations; the descriptive Berry comparison (§5.5) shows this convergence in Snyder but not in the comparison poet, but with the between-poet differences unestablished it adds no inferential weight here. The perceptual dimension changes over the same career span but is partly shared with long poetic careers; this overall pattern is consistent with Dahl et al.’s (2015) account of integrated practice as spanning multiple dimensions.

### 5.5 The Berry comparison: shared aging versus practice-related change

Applying the same indicators and statistical procedures to Berry’s 280 poems yields a sharp descriptive contrast (Table 2; the full two-framework comparison appears in Supplementary Appendix G, Table G-1). On the dimensions central to the meditation hypothesis, Berry’s language is essentially flat across his half-century career: negative-emotion frequency (τ = −0.055, n.s.), first-person pronouns (τ = +0.001, n.s.), the certainty-to-tentativeness ratio (τ = −0.065, n.s.), and mean syllables per word (τ = +0.054, n.s.) show no significant trend–whereas all four change significantly in Snyder. Because Berry is simultaneously aging and writing within the same nature-oriented register, this descriptive contrast is the pattern a practice-related interpretation of the affective settling and first-person/epistemic attenuation would predict; on its own, however, it neither tests nor excludes aging- or genre-based alternatives. The between-poet contrast itself, however, must be tested rather than inferred from this pattern of significance. We therefore estimated τ(Snyder) −τ(Berry) for every Table 2 indicator with a collection-blocked bootstrap (*B* = 20,000; §4.5): under the available (asymmetric) temporal indices, all pre-specified percentile 95% confidence intervals include zero (Table 2), so no between-poet difference is established for any indicator; with only nine and eleven collections as resampling units these intervals are necessarily wide, and failure to establish a difference is accordingly not evidence that the two poets’ trends are equal. For negative-emotion frequency and mean syllables per word the differences are sizable and in the predicted direction (Δτ = −0.113 and +0.134), and for these two indicators the conclusion is sensitive to the interval construction: the pre-specified percentile intervals include zero, basic intervals exclude it for both, and the standard BCa interval excludes zero for negative-emotion frequency while its lower endpoint for mean syllables per word is numerically indistinguishable from zero (Supplementary Appendix G); we therefore treat these two contrasts as suggestive but not established. For first-person pronouns and the certainty-to-tentativeness ratio–whose coefficients are close (−0.085 vs. −0.065)–no difference is indicated under any construction. Throughout this article, “practice-consistent” is therefore a descriptive label for the within-poet pattern, and no inferential conclusion rests on the between-poet distinction. On the perceptual dimension, however, the two poets converge: embodied and sensory vocabulary thin significantly in Berry as well (somatic τ = −0.210, atmospheric τ = −0.205, total sensory τ = −0.170, tactile τ = −0.121, nature-to-self subject ratio τ = −0.180; all *p* < 0.05), showing that sensory thinning is not unique to Snyder and may reflect processes shared across long poetic careers rather than a uniquely Zen pattern. The comparison is thus descriptively consistent with a practice-related reading of the affective and self-referential dimensions–subject to the inferential limits just stated–and prompts a principled narrowing of the perceptual claim (§6.2).

## 6 Discussion

### 6.1 Evidence supporting H1, H2, and H3

The results are broadly consistent with the three hypotheses, though the strength and specificity of the evidence differ across dimensions. Support is strongest for the affective dimension (H1: the monotonic within-corpus decline in negative-emotion vocabulary, with no counterpart trend in the matched non-practicing poet–descriptively the sharpest contrast, though the between-poet difference itself is not statistically established, §5.5); the self-referential dimension is supported with qualifications (H2: the within-corpus declines in first-person pronouns and cognitive certainty have no significant counterpart in Berry, but the between-poet differences are likewise not established, and the two indicators differ in robustness, §5.2 and §6.1 below); and the evidence is mixed for the perceptual dimension (H3: the rise in lexical abstraction has no counterpart trend in Berry, whereas the thinning of sensory vocabulary–and the parallel shift in grammatical subject–appears in both poets). None of the rivals considered here is fully excluded by the present observational design. The Berry comparison contributes asymmetrically: it shows that the perceptual pattern overlaps substantially with another long poetic career, which counts against treating that pattern as distinctive to Snyder, whereas for the affective and self-referential changes the sample pattern is descriptive only and does not narrow the space of alternatives.

The data support H1: all three frameworks confirm the monotonic decline of negative-emotion vocabulary, with no significant linear trend in positive or neutral categories. This asymmetry–decline in the negative categories without a detectable trend in the positive and neutral ones–does not support the affective-flattening rival hypothesis and is consistent with the *ānxīn* mechanism–the settling, rather than suppression, of affect.

The data support H2, though not uniformly across its four indicators. First-person pronouns, somatic vocabulary, the nature-to-self subject ratio, and certainty vocabulary all decline over the career; of these, the declines in first-person pronouns and in certainty vocabulary have no significant counterpart in the comparison poet and are therefore descriptively consistent with a practice-related interpretation (the between-poet differences are not established, §5.5), whereas the declines in somatic vocabulary and in the subject ratio are shared with Berry (Table 2) and are better read as features of a long poetic career. The two carrying indicators also differ in robustness and should not be weighed equally: first-person pronouns are significant in all three analytical frameworks but their collection-blocked bootstrap CI spans zero (Table 1, †); the certainty-to-tentativeness ratio is significant in the continuous and endpoint frameworks (A and C) but not at the collection level (B, *n* = 9), and therefore does not meet the 3/3 criterion for inclusion in Table 1 (full three-framework disclosure in Supplementary Appendix C, Table C-4). The nature-to-self ratio, moreover, declines because nature subjects recede rather than because self subjects do. The de-centering evidence is thus narrower than a simple four-indicator convergence would suggest, and the pronoun and certainty results carry the weight of the anātman interpretation. This pattern does not support the stylistic-maturation rival hypothesis: the data instead show pronoun decline accompanied by increasing syntactic ellipsis rather than complexity. Notably, the reduction in certainty assertions and the increase in tentativeness reveal an additional facet of H2: the dissolution of self manifests not only in the retreat of “I” but in a shift of cognitive stance from assertion toward openness–a dimension seldom foregrounded in doctrinal commentary.

The data support H3: embodied sensory specificity thins significantly in T3, with tactile vocabulary showing the steepest decline; mean syllables per word rises monotonically (τ = +0.188), consistent with a shift in perceptual organization toward greater abstraction. In the descriptive Berry comparison (§5.5), the rise in abstraction has no counterpart in Berry, whereas sensory thinning is observed in both poets; we therefore treat the perceptual dimension as a mixed case rather than a wholly practice-driven one.

Career-long trends appear across all three theoretical domains rather than in a single isolated measure. The changepoint evidence is more limited: its coincident locations at pen = 10 concern one affective indicator and two self-related indicators, and the H3 indicator included in the five-series PELT check (mean syllables per word) yields no breakpoint. The broader multidomain pattern constitutes the core contribution of this study and brings the findings into dialogue with the Dahl-Lutz-Davidson three-family framework. The sixty-year temporal scope confers distinctive value. The laboratory studies that anchor this literature observe practitioners over weeks or months (Davidson et al., 2003), and critical reviews of the field have identified insufficient temporal depth as a standing limitation (Van Dam et al., 2018); the present corpus spans six decades, extending the observational window well beyond what such designs afford.

### 6.2 Alternative explanations

The aging hypothesis. The most direct competing explanation is natural aging, with which Snyder’s practice years are collinear; socioemotional selectivity theory, in particular, predicts age-related shifts in emotional experience and goals across adulthood (Carstensen et al., 1999). The Berry analysis (§5.5) provides a targeted comparison rather than a control that isolates practice: a non-practicing poet matched to Snyder on era and theme, aging across a comparable span, shows no significant decline in negative-emotion vocabulary, first-person pronouns, or epistemic certainty. This descriptive contrast is the pattern a practice-related interpretation of the affective and self-referential changes would predict, though the between-poet differences themselves are not statistically established (§5.5). At the same time, Berry’s language also thins in sensory and somatic vocabulary, so the shared component is consistent with processes common to long poetic careers rather than uniquely practice-related. The comparison therefore bears on the aging alternative asymmetrically: the shared sensory and somatic thinning counts against treating that component as distinctive to practice, whereas for the affective and self-referential changes–where the between-poet differences are not established–it does not narrow the aging alternative.

The period aesthetic hypothesis. The broader Beat Generation context may account for the emotional intensity of the early work. Yet not all Beat poets underwent a comparable transformation: Kerouac’s late years were marked by alcoholism and despair (Charters, 1973), and Ginsberg retained fierce political anger into his late work (Miles, 2000). Snyder’s within-corpus trajectory–particularly the settling of affect and attenuation of self–still requires explanation; because the corresponding between-poet differences are not established, however, the descriptive Berry pattern does not distinguish the period-aesthetic account from a practice-related one.

The reverse-causality hypothesis. A further alternative is that Snyder may have treated poetic composition itself as a form of practice, meaning the linguistic changes could be the medium of practice rather than a record of its effects. This possibility cannot be fully excluded, but three considerations argue against it as the primary account. First, Snyder arrived in Kyoto in 1956 and began formal Rinzai training before the publication of any of the analyzed collections–and his documented zazen practice reaches back to the earliest analyzed poems (§4.2); zazen, kōan work, and monastic duties are not the same activity as poetic composition (Suiter, 2002; Snyder, 1999). Second, biographical research documents sustained daily zazen and annual sesshins over the following decades (Martin and Snyder, 2014), activities independent of poetic production. Third, even if composition partially served a practice function, it does not explain why the earlier detected changepoint falls close to a documented transition in the setting and form of practice rather than at a peak of compositional intensity. A more precise formulation is that practice and poetry may have been bidirectionally coupled, but pure reverse causality cannot account for the observed overall structure.

### 6.3 Limitations and future directions

As a single-case study, this work’s generalizability is necessarily constrained, and chronological age and years of practice are collinear in this design. The Berry analysis should be understood as a targeted comparative probe–a most-similar comparative case rather than a control condition. Its evidential status is descriptive: the primary percentile tests establish no reliable between-poet difference for any indicator (§5.5), and the comparison additionally relies on a coarser temporal index for Berry (volume publication years rather than poem-level composition dates) and on an author-curated retrospective corpus, both of which limit the precision and comparability of the Berry trend estimates (§4.5). A single, purposively selected comparison poet cannot break the collinearity among practice, chronological age, historical period, career stage, and life circumstances, nor can the two poets be assumed to differ only in the variable of interest; the comparison bears on the alternatives only asymmetrically–trends that also appear in the comparison poet count against treating a change as distinctive to Snyder, whereas trends observed in Snyder alone, with the between-poet difference unestablished, do not narrow the space of alternative explanations–and it cannot eliminate selection effects; a definitive separation of practice from aging would require a larger, non-selectively sampled panel of practicing and non-practicing poets analyzed in a regression framework, which we identify as the essential next step. We have taken a first step in this direction with the Berry comparison (§5.5); extending it–to further non-practicing contemporaries and to other long-term practitioner-poets, and ultimately to a larger, non-selectively sampled panel analyzed in a regression framework–is the most immediate next step for strengthening the decoupling of age and practice.

Frequency-based methods capture distributional patterns but cannot fully address metaphor and irony in poetic language–a poem may convey intense negative affect through figurative constructions while containing few standard negative-emotion words. The convergence of the dictionary-based affective signal with an independent lexicon (Empath) and with non-lexical syntactic indicators makes it unlikely that the pattern is an artifact of any single lexicon’s treatment of figurative language. Future work incorporating semantically aware or metaphor-sensitive NLP tools (such as SentiArt; Jacobs, 2019) and vector semantic models would complement this study’s approach with greater granularity.

The observational nature of the data keeps causal inference constrained. The temporal precedence of practice and the earlier changepoint’s proximity to a documented change in practice setting together constitute longitudinal observational rather than strictly experimental evidence; whether practice shaped language, or life circumstances shaped both simultaneously, cannot be definitively resolved within this design. Addressing this limitation awaits rigorously controlled comparative studies across practitioners. A further, related limitation concerns the material itself: poetry is a highly edited, aesthetic, and culturally situated form of language, so the present indicators capture linguistic expression rather than psychological states directly, and the inferences drawn from them are correspondingly bounded. A cross-linguistic comparison with Chinese Zen poetry offers a natural extension; because it must contend with the comparability of very different historical and literary contexts and with the construction of a comparable corpus, we regard it as a valuable direction for dedicated future study rather than one to be undertaken here.

As a methodological extension, this analytical framework could in principle be generalized across traditions to other practitioners with rich textual legacies–Matsuo Bashō in the Japanese haiku tradition, Rumi in the Sufi tradition, Milarepa in Tibetan Buddhism–examining whether different practice paths leave common or tradition-specific linguistic traces.

## 7 Conclusion

This study asked whether long-term meditation leaves measurable traces in the practitioner’s natural language. Computational textual analysis of nine poetry collections spanning Gary Snyder’s six-decade career reveals career-long changes across three analytical domains–emotion regulation, self-reference, and perceptual organization. A descriptive comparison with a non-practicing poet (Berry) shows the affective and self-referential trends in Snyder but not in Berry–the between-poet differences themselves are not established (§5.5)–while part of the perceptual pattern appears in both poets and may reflect processes shared across long poetic careers.

At the methodological level, this work opens a new empirical pathway for meditation science. Naturally produced creative texts, as a non-reactive observational dimension, can capture the deep automatized linguistic patterns that self-report and neuroimaging struggle to reach, while a sixty-year temporal scope extends far beyond the observational reach of existing meditation research. Together, these two features allow the long-term psychological evolution of deep practitioners–long marked as unknown territory–to be approached with an empirical instrument commensurate with its timescale. Within Snyder scholarship, the qualitative consensus that deepening practice accompanies linguistic transformation finds its first quantitative evidence, moving this research tradition onto a track amenable to replication and verification. At the interdisciplinary level, this study brings the psychology of meditation, computational literary studies, and Snyder scholarship into a shared evidential framework.

As a hypothesis-generating single-case study, the core proposition advanced here is this: the corpus contains career-long changes across affective, self-referential, and perceptual language measures that are descriptively consistent with, but do not prove, a practice-related interpretation; the earlier of the two detected changepoints falls close to a documented transition in the setting and form of practice. This analytical framework can in principle be extended to other practitioner-poets with systematic textual records, to other contemplative traditions, and to other longitudinal archives in which behavioral records are produced without deliberate self-presentation. The cross-case comparative analysis and methodological deepening discussed above represent the critical directions for testing this proposition and extending this framework.

Implications and recommendations. These findings carry implications for three fields. For meditation science, naturally produced text offers a non-reactive, decades-spanning measure that complements self-report and neuroimaging, which are confined to short windows and reactive instruments; we recommend the systematic construction of longitudinal textual corpora for documented long-term practitioners. For computational literary studies, the results show that distributional features can index an author’s evolving cognitive-affective schemata, not merely surface style, motivating schema-level rather than purely stylistic distant reading. For the study of religion and contemplative traditions, the approach provides an empirical handle on transformation claims long deemed “ineffable,” without reducing the experience itself–measuring the traces rather than the content of practice. Concretely, we recommend three next steps: (1) cross-case comparison against non-practicing contemporaries and other practitioner-poets to decouple practice from aging; (2) extension to other contemplative traditions with rich textual legacies (Bashō, Rumi, Milarepa); and (3) integration of metaphor-aware and vector-semantic NLP–including recent large-language-model-based approaches–to capture figurative affect that frequency methods miss.

## Data Availability

The original contributions presented in the study are included in the article/Supplementary material, further inquiries can be directed to the corresponding author/s.

## References

[B1] Almon B. (1979). *Gary Snyder.* Boise: Boise State University.

[B2] Bargh J. A. (1994). “The Four Horsemen of Automaticity: Awareness, Intention, Efficiency, and Control in Social Cognition,” in *Handbook of Social Cognition*, 2nd Edn, eds Wyer R. S. Srull T. K. (Hillsdale: Erlbaum), 1–40.

[B3] Barnhill D. L. (1997). “Great Earth Sangha: Gary Snyder’s View of Nature as Community,” in *Buddhism and Ecology: The Interconnection of Dharma and Deeds*, eds Tucker M. E. Williams D. R. (Cambridge: Harvard University Press), 187–217.

[B4] Berry W. (2012). *New Collected Poems.* Berkeley: Counterpoint.

[B5] Bi J. (2023). Symbiotic nurture between literature, culture and nature in Gary Snyder’s meta-picto-poetry of landscape. *Trans. Form. Ação* 46, 163–182.

[B6] Buswell R. E. Lopez D. S. Jr. (2014). *The Princeton Dictionary of Buddhism.* Princeton: Princeton University Press.

[B7] Carstensen L. L. Isaacowitz D. M. Charles S. T. (1999). Taking time seriously: a theory of socioemotional selectivity. *Am. Psychol*. 54, 165–181. 10.1037//0003-066x.54.3.165 10199217

[B8] Charters A. (1973). *Kerouac: A Biography.* San Francisco: Straight Arrow Books.

[B9] Dahl C. J. Lutz A. Davidson R. J. (2015). Reconstructing and deconstructing the self: Cognitive mechanisms in meditation practice. *Trends Cogn Sci*. 19, 515–523. 10.1016/j.tics.2015.07.001 26231761 PMC4595910

[B10] Davidson R. J. Kabat-Zinn J. Schumacher J. Rosenkranz M. Muller D. Santorelli S. F. et al. (2003). Alterations in brain and immune function produced by mindfulness meditation. *Psychosom. Med*. 65, 564–570. 10.1097/01.psy.0000077505.67574.e3 12883106

[B11] De Sisto M. Hernández-Lorenzo L. De la Rosa J. Ros S. González-Blanco E. (2024). Understanding poetry using natural language processing tools: A survey. *Dig. Scholar. Hum.* 39, 500–521. 10.1093/llc/fqae001

[B12] Dean T. (1991). *Gary Snyder and the American Unconscious: Inhabiting the Ground.* New York: St. Martin’s Press.

[B13] Ehmann S. Sezer I. Treves I. N. Gabrieli J. D. E. Sacchet M. D. (2025). Mindfulness, cognition, and long-term meditators: Toward a science of advanced meditation. *Imag. Neurosci*. 3:IMAGa82. 10.1162/IMAG.a.82 40800948 PMC12330868

[B14] Fast E. Chen B. Bernstein M. S. (2016). “Empath: Understanding Topic Signals in Large-Scale Text,” in *Proceedings of the 2016 CHI Conference on Human Factors in Computing Systems*, (New York: ACM), 4647–4657.

[B15] Flyvbjerg B. (2006). Five misunderstandings about case-study research. *Q. Inquiry* 12, 219–245.

[B16] Gerring J. (2007). *Case Study Research: Principles and Practices.* Cambridge: Cambridge University Press.

[B17] Goleman D. Davidson R. J. (2017). *Altered Traits: Science Reveals How Meditation Changes Your Mind, Brain, and Body.* New York: Avery.

[B18] Gray T. (2006). *Gary Snyder and the Pacific Rim: Creating Countercultural Community.* Iowa City: University of Iowa Press.

[B19] Grimmer J. Roberts M. E. Stewart B. M. (2022). *Text as Data: A New Framework for Machine Learning and the Social Sciences.* Princeton: Princeton University Press.

[B20] Hölzel B. K. Lazar S. W. Gard T. Schuman-Olivier Z. Vago D. R. Ott U. (2011). How does mindfulness meditation work? Proposing mechanisms of action from a conceptual and neural perspective. *Perspect Psychol Sci*. 6, 537–559. 10.1177/1745691611419671 26168376

[B21] Hunt A. (2004). *Genesis, Structure, and Meaning in Gary Snyder’s Mountains and Rivers Without End.* Reno: University of Nevada Press.

[B22] Jacobs A. M. (2015). Neurocognitive poetics: methods and models for investigating the neuronal and cognitive-affective bases of literature reception. *Front Hum Neurosci*. 9:186. 10.3389/fnhum.2015.00186 25932010 PMC4399337

[B23] Jacobs A. M. (2019). Sentiment analysis for words and fiction characters from the perspective of computational (neuro-)poetics. *Front. Robot AI*. 6:53. 10.3389/frobt.2019.00053 33501068 PMC7805775

[B24] Kahneman D. (2011). *Thinking, Fast and Slow.* New York: Farrar, Straus and Giroux.

[B25] Kao J. Jurafsky D. (2012). “A Computational Analysis of Style, Affect, and Imagery in Contemporary Poetry,” in *Proceedings of the NAACL-HLT 2012 Workshop on Computational Linguistics for Literature*, (Montréal: Association for Computational Linguistics), 8–17.

[B26] Killick R. Fearnhead P. Eckley I. A. (2012). Optimal detection of changepoints with a linear computational cost. *J. Am. Statis. Ass.* 107, 1590–1598.

[B27] Kim E. Klinger R. (2019). A survey on sentiment and emotion analysis for computational literary studies. *Zeitschrift. Für. Digitale. Geisteswissenschaften* 10.17175/2019_008 32015775

[B28] Krippendorff K. (2018). *Content Analysis: An Introduction to Its Methodology*, 4th Edn. Los Angeles: SAGE.

[B29] Landis J. R. Koch G. G. (1977). The measurement of observer agreement for categorical data. *Biometrics* 33, 159–174. 843571

[B30] Laukkonen R. E. Slagter H. A. (2021). From many to (n)one: Meditation and the plasticity of the predictive mind. *Neurosci. Biobehav. Rev*. 128, 199–217. 10.1016/j.neubiorev.2021.06.021 34139248

[B31] Lutz A. Slagter H. A. Dunne J. D. Davidson R. J. (2008). Attention regulation and monitoring in meditation. *Trends Cogn. Sci*. 12, 163–169. 10.1016/j.tics.2008.01.005 18329323 PMC2693206

[B32] Mar R. A. Oatley K. (2008). The function of fiction is the abstraction and simulation of social experience. *Perspect. Psychol. Sci*. 3, 173–192. 10.1111/j.1745-6924.2008.00073.x 26158934

[B33] Martin J. Snyder G. (2014). *Nobody Home: Writing, Buddhism, and Living in Places.* San Antonio: Trinity University Press.

[B34] Miall D. S. Kuiken D. (1994). Foregrounding, defamiliarization, and affect: response to literary stories. *Poetics* 22, 389–407.

[B35] Miles B. (2000). *Allen Ginsberg: A Biography.* London: Virgin Books.

[B36] Mohammad S. M. Turney P. D. (2013). Crowdsourcing a word–emotion association lexicon. *Comp. Intell.* 29, 436–465.

[B37] Murphy P. D. (1990). *Critical Essays on Gary Snyder.* Boston: G. K. Hall.

[B38] Murphy P. D. ed. (1992). *Understanding Gary Snyder.* Columbia: University of South Carolina Press.

[B39] Murphy P. D. (2000). *A Place for Wayfaring: The Poetry and Prose of Gary Snyder.* Corvallis: Oregon State University Press.

[B40] Öhman E. Bizzoni Y. Feldkamp Moreira P. Nielbo K. L. (2024). “EmotionArcs: Emotion Arcs for 9,000 Literary Texts,” in *Proceedings of the 8th Joint SIGHUM Workshop on Computational Linguistics for Cultural Heritage, Social Sciences, Humanities and Literature (LaTeCH-CLfL 2024)*, (St. Julians: Association for Computational Linguistics), 51–66.

[B41] Pargament K. I. (2002). The Bitter and the sweet: An Evaluation of the costs and benefits of religiousness. *Psychol. Inq.* 13, 168–181.

[B42] Pennebaker J. W. (2011). *The Secret Life of Pronouns: What Our Words Say About Us.* New York: Bloomsbury Press.

[B43] Pennebaker J. W. Stone L. D. (2003). Words of wisdom: language use over the life span. *J. Pers. Soc. Psychol*. 85, 291–301. 10.1037/0022-3514.85.2.291 12916571

[B44] Reagan A. J. Mitchell L. Kiley D. Danforth C. M. Dodds P. S. (2016). The Emotional Arcs of Stories Are Dominated by Six Basic Shapes. *EPJ Data Science* 5, 31.

[B45] Rebora S. (2023). Sentiment analysis in literary studies: A critical survey. *Dig. Hum. Q.* 17:000691.

[B46] Sacchet M. D. Fava M. Garland E. L. (2024). Modulating self-referential processing through meditation and psychedelics: is scientific investigation of self-transcendence clinically relevant? *World Psychiatry*. 23, 298–299. 10.1002/wps.21214 38727064 PMC11083968

[B47] Schneider D. (2015). *Crowded by Beauty: The Life and Zen of Poet Philip Whalen.* Oakland: University of California Press.

[B48] Snyder G. (1999). *The Gary Snyder Reader: Prose, Poetry, and Translations, 1952–1998.* Washington, DC: Counterpoint.

[B49] Suiter J. (2002). *Poets on the Peaks: Gary Snyder, Philip Whalen & Jack Kerouac in the North Cascades.* Washington, DC: Counterpoint.

[B50] Tang Y. Y. Hölzel B. K. Posner M. I. (2015). The neuroscience of mindfulness meditation. *Nat Rev Neurosci*. 16, 213–225. 10.1038/nrn3916 25783612

[B51] Tausczik Y. R. Pennebaker J. W. (2010). The Psychological meaning of words: LIWC and computerized text analysis methods. *J Lang SocPsychol* 29, 24–54.

[B52] Underwood T. (2019). *Distant Horizons: Digital Evidence and Literary Change.* Chicago: University of Chicago Press.

[B53] Van Dam N. T. van Vugt M. K. Vago D. R. Schmalzl L. Saron C. D. Olendzki A. et al. (2018). Mind the Hype: A critical evaluation and prescriptive agenda for research on mindfulness and meditation. *Perspect Psychol. Sci*. 13, 36–61. 10.1177/1745691617709589 29016274 PMC5758421

[B54] Wirth J. M. (2017). *Mountains, Rivers, and the Great Earth: Reading Gary Snyder and Dōgen in an Age of Ecological Crisis.* Albany: State University of New York Press.

[B55] Yamazato K. (1990). “How to Be in This Crisis: Gary Snyder’s Cross-Cultural Vision in Turtle Island,” in *Critical Essays on Gary Snyder*, ed. P. D. Murphy (Boston: G. K. Hall), 230–246.

[B56] Yin R. K. (2018). *Case Study Research and Applications: Design and Methods*, 6th Edn. Thousand Oaks: Sage.

