# Supplementary material for: Sixty years of linguistic change in the poetry of a Zen practitioner: a longitudinal case study of Gary Snyder
Source: Front Psychol. 2026 Sep 25;17:1879502. doi: 10.3389/fpsyg.2026.1879502 (PMC13649949; doi:10.3389/fpsyg.2026.1879502)
Supplement: Supplementary file 1 [file Supplementary_file_1.zip › Supplementary Materials/Appendix.docx]

**Appendix: Detailed Statistical Results**

# **Appendix A. Corpus Composition**

**Table A-1. Basic information for the nine poetry collections, with period assignment.**

| **Collection** | **Year** | **Composition years** | **Years of practice** | **Total poems** | **T1** | **T2** | **T3** |
| --- | --- | --- | --- | --- | --- | --- | --- |
| *Riprap* | 1959 | 1953–1958 | −3 to 2 | 23 | 23 | — | — |
| *Myths & Texts* | 1960 | 1951–1956 | −5 to 0 | 48 | 48 | — | — |
| *The Back Country* | 1968 | 1953–1965 | −3 to 9 | 86 | 86 | — | — |
| *Regarding Wave* | 1970 | 1966–1969 | 10 to 13 | 61 | 61 | — | — |
| *Turtle Island*† | 1974 | 1968–1974 | 12 to 18 | 63 | 7 | 56 | — |
| *Axe Handles*† | 1983 | 1969–1982 | 13 to 26 | 72 | 2 | 70 | — |
| *Mountains and Rivers Without End*† | 1996 | 1956–1991 | 0 to 35 | 39 | 9 | 19 | 11 |
| *Danger on Peaks* | 2004 | 1996–2003 | 40 to 47 | 78 | — | — | 78 |
| *This Present Moment* | 2015 | 2007–2013 | 51 to 57 | 47 | — | — | 47 |
| **Total** |  |  |  | **517** | **236** | **145** | **136** |

*Note.* Collections marked with † span more than one period; individual poems are assigned to the corresponding period by actual composition date (see Tables A-2 and A-3). Twenty-four Hanshan translations and eighteen Miyazawa Kenji translations have been excluded. Years of practice are computed from 1956—the year Snyder departed for Japan to begin formal Zen training—using each poem’s composition date. T1 (years of practice −5 to 14; its 57 earliest poems, composed 1951–1955, precede the 1956 onset of formal training): 236 poems; T2 (15–27 years): 145 poems; T3 (28+ years): 136 poems.

**Table A-2. Composition dates and period assignment for the 39 poems in *Mountains and Rivers Without End*.**

Although the volume was published in 1996, the poems were composed between 1956 and 1991. Each poem is assigned to a period by its actual composition date.

| **Composition years** | **Years of practice** | **Period** | **Number of poems** | **Representative titles** |
| --- | --- | --- | --- | --- |
| 1956 | 0 | T1 | 1 | “Endless Streams and Mountains” |
| 1957 | 1 | T1 | 1 | “Night Highway 99” |
| 1965 | 9 | T1 | 2 | “Jackrabbit”; “The Elwha River” |
| 1966–1968 | 10–12 | T1 | 3 | “Three Worlds…”; “Walking the New York Bedrock”; “Bubbs Creek Haircut”, etc. |
| 1970 | 14 | T1 | 2 | “The Hump-backed Flute Player”; “The Market” |
| **Subtotal** |  | **T1** | **9 (23%)** |  |
| 1974–1983 | 18–27 | T2 | 19 | “Raven’s Beak River”; “The Blue Sky”; “The Canyon Wren”, etc. |
| **Subtotal** |  | **T2** | **19 (49%)** |  |
| 1984–1991 | 28–35 | T3 | 11 | “Cross-Legg’d”; “We Wash Our Bowls…”; “Finding the Space in the Heart”, etc. |
| **Subtotal** |  | **T3** | **11 (28%)** |  |

*Note.* Composition dates are based on documentary evidence in the secondary literature (Hunt, 2004; Snyder, 1999); see phase0_date_matching.py for assignment details. Period assignment by composition date ensures that the years-of-practice value associated with each poem accurately reflects the practice stage at the time of composition.

**Table A-3. Composition dates and period assignment for the 63 poems in *Turtle Island*.**

The volume was published in 1974; the poems were composed between 1968 and 1974. Seven poems are assigned to T1 and the remaining 56 to T2.

| **Composition years** | **Years of practice** | **Period** | **Number of poems** |
| --- | --- | --- | --- |
| 1968 | 12 | T1 | 1 |
| 1969 | 13 | T1 | 3 |
| 1970 | 14 | T1 | 3 |
| **Subtotal** |  | **T1** | **7 (11%)** |
| 1971–1973 | 15–17 | T2 | 55 |
| 1974 | 18 | T2 | 1 |
| **Subtotal** |  | **T2** | **56 (89%)** |

*Note.* The 55 poems composed during 1971–1973 correspond to 15–17 years of practice and sit at the T1/T2 boundary; assigning them to T2 is consistent with the T2 practice range of 15–27 years.

# **Appendix B. Complete Results: NRC Emotion Lexicon Analysis**

**Table B-1. Period means (per thousand words) for the ten NRC emotion categories.**

| **Emotion category** | **T1** | **T2** | **T3** | **Peak period** | **KW statistic** | **KW *p*** |
| --- | --- | --- | --- | --- | --- | --- |
| negative | 43.15 | 38.46 | 35.95 | T1 | 23.557 | <.001*** |
| disgust | 14.63 | 13.13 | 10.68 | T1 | 9.186 | .010** |
| surprise | 14.08 | 10.88 | 12.64 | T1 | 7.652 | .022* |
| sadness | 21.51 | 20.55 | 18.44 | T1 | 6.325 | .042* |
| anger | 16.56 | 13.98 | 14.63 | T1 | 6.153 | .046* |
| fear | 20.06 | 18.25 | 20.12 | T1 | 4.870 | .088(†) |
| trust | 24.47 | 27.80 | 28.07 | T3 | 3.252 | .197 |
| joy | 23.26 | 21.15 | 20.78 | T1 | 1.624 | .444 |
| positive | 48.01 | 50.96 | 47.76 | T2 | 0.830 | .660 |
| anticipation | 21.11 | 21.15 | 24.50 | T3 | 1.742 | .418 |

*Note.* Kruskal-Wallis test, *df* = 2. *p < .05, **p < .01, ***p < .001; (†) marginal.

**Table B-2. Three-framework robustness checks for the NRC emotion categories.**

| **Emotion category** | **Framework A: Kendall τ (*n* = 517)** | **Framework B: τ (*n* = 9)** | **Framework C: rank-biserial** | **Median change (%)** | **Frameworks** |
| --- | --- | --- | --- | --- | --- |
| negative | −0.169*** | −0.722** | −0.255*** | −33.2% | **3/3** |
| disgust | −0.113*** | −0.444 | −0.161** | −37.3% | 2/3 |
| sadness | −0.101** | −0.611* | −0.130* | −27.4% | **3/3** |
| anger | −0.094** | −0.611* | −0.108(†) | −30.4% | 2/3 |
| fear | −0.088** | −0.500(†) | −0.100 | −16.3% | 1/3 |
| surprise | −0.061* | −0.333 | −0.065 | −12.4% | 1/3 |
| joy | −0.046 | −0.278 | −0.068 | −2.3% | 0/3 |
| positive | −0.001 | −0.278 | +0.016 | +2.3% | 0/3 |
| trust | +0.021 | +0.222 | +0.075 | +17.5% | 0/3 |
| anticipation | −0.015 | 0.000 | +0.023 | +6.7% | 0/3 |

*Note.* Framework A: Kendall rank correlation between each indicator and years of practice as a continuous variable (*n* = 517). Framework B: collection-level means, Kendall rank correlation (*n* = 9). Framework C: Mann-Whitney U test for T1 vs. T3, with rank-biserial correlation as effect-size measure. (†) .05 < *p* < .10.

# **Appendix C. Complete Results: Multi-Level Indicators of Self-Effacement**

**Table C-1. Three-framework tests for first-person pronoun and somatic vocabulary indicators.**

| **Indicator** | **Framework A: τ (*n* = 517)** | **Framework B: τ (*n* = 9)** | **Framework C: *r*b** | **Median change (%)** | **Frameworks** |
| --- | --- | --- | --- | --- | --- |
| I/me/my pronoun frequency | −0.086** | −0.588* | −0.124* | −72.9% | **3/3** |
| Somatic word frequency (`body_rate`) | −0.158*** | −0.667* | −0.344*** | −77.3% | **3/3** |
| Somatic-to-nature word ratio | −0.127*** | −0.611* | −0.283*** | −70.6% | **3/3** |
| Embodied sensory word frequency | −0.115*** | −0.778** | −0.192** | −50.4% | **3/3** |

**Table C-2. Collection-level means by syntactic subject type.**

| **Collection** | **Year** | **Period** | **Self-subject rate** | **Human-subject rate** | **Nature-subject rate** | **Impersonal-subject rate** | Nature-to-self ratio |
| --- | --- | --- | --- | --- | --- | --- | --- |
| *Riprap* | 1959 | T1 | 0.111 | 0.210 | 0.072 | 0.038 | 0.592 |
| *Myths & Texts* | 1960 | T1 | 0.054 | 0.286 | 0.090 | 0.037 | 0.635 |
| *The Back Country* | 1968 | T1 | 0.040 | 0.245 | 0.080 | 0.021 | 0.615 |
| *Regarding Wave* | 1970 | T1 | 0.010 | 0.237 | 0.131 | 0.015 | 0.596 |
| *Turtle Island* | 1974 | T2 | 0.037 | 0.226 | 0.105 | 0.051 | 0.618 |
| *Axe Handles* | 1983 | T2 | 0.054 | 0.211 | 0.065 | 0.020 | 0.265 |
| *Mountains and Rivers Without End* | 1996 | T3 | 0.059 | 0.195 | 0.042 | 0.017 | 0.227 |
| *Danger on Peaks* | 2004 | T3 | 0.046 | 0.256 | 0.077 | 0.014 | 0.380 |
| *This Present Moment* | 2015 | T3 | 0.037 | 0.271 | 0.042 | 0.043 | 0.110 |

**Table C-3. Three-framework tests for syntactic subject types.**

| **Indicator** | **Framework A: τ (*n* = 517)** | **Framework B: τ (*n* = 9)** | **Framework C: *r*b** | **Frameworks** |
| --- | --- | --- | --- | --- |
| Self-subject rate | +0.008 | −0.222 | +0.018 | 0/3 |
| Human-subject rate | −0.023 | 0.000 | −0.030 | 0/3 |
| Nature-subject rate | −0.119*** | −0.389 | −0.158** | 2/3 |
| Impersonal-subject rate | −0.013 | −0.278 | −0.008 | 0/3 |
| Nature-to-self subject ratio | **−0.130***** | **−0.556*** | **−0.168**** | **3/3** |

**Table C-4. Three-framework tests for cognitive-stance indicators.**

| **Indicator** | **Framework A: τ (*n* = 517)** | **Framework B: τ (*n* = 9)** | **Framework C: *r*b** | **Frameworks** |
| --- | --- | --- | --- | --- |
| Certainty word frequency | −0.049 | −0.085 | −0.124* | 1/3 |
| Tentativeness word frequency | +0.065* | +0.262 | +0.132* | 2/3 |
| **Certainty-to-tentativeness ratio** | **−0.085**** | **−0.423** | **−0.181**** | **2/3** |

# **Appendix D. Complete Results: Perceptual Transformation (Ānimitta)**

**Table D-1. Period means (per thousand words) for the five-sense vocabulary categories.**

| **Sensory category** | **T1** | **T2** | **T3** | **Peak period** | **Trend direction** |
| --- | --- | --- | --- | --- | --- |
| Visual words | 17.22 | 21.35 | 17.30 | T2 | n.s. |
| Auditory words | 4.75 | 8.26 | 4.24 | T2 | n.s. |
| Tactile words | 13.92 | 14.19 | 9.27 | T2 | ↓*** |
| Olfactory words | 2.39 | 2.60 | 2.46 | T2 | n.s. |
| Total sensory word frequency | 42.28 | 51.41 | 35.95 | T2 | ↓* |
| Sensory evenness | — | — | — | — | ↓*** |

**Table D-2. Three-framework tests for the perceptual-dimension core indicators.**

| **Indicator** | **Framework A: τ (*n* = 517)** | **Framework B: τ (*n* = 9)** | **Framework C: *r*b** | **Median change (%)** | **Frameworks** |
| --- | --- | --- | --- | --- | --- |
| **Tactile word frequency** | **−0.129*** | **−0.761*** | **−0.235*** | **−73.9%** | **3/3** |
| **Total sensory word frequency** | **−0.077*** | **−0.611*** | **−0.183*** | **−25.0%** | **3/3** |
| **Sensory evenness** | **−0.119***** | **−0.761*** | **−0.209***** | **−26.1%** | **3/3** |
| Visual word frequency | −0.056(†) | −0.278 | −0.074 | −13.4% | 0/3 |
| Auditory word frequency | −0.035 | −0.471 | −0.026 | — | 0/3 |

*Note.* Visual and auditory categories show no significant linear trend, indicating that the T3 sensory thinning is not uniform across modalities but selectively concentrated on touch. Olfactory vocabulary, with extremely low base rates (T1 mean = 1.66‰), is excluded from the three-framework testing.

**Table D-3. Three-framework tests for word-morphology indicators.**

| **Indicator** | **Framework A: τ (*n* = 517)** | **Framework B: τ (*n* = 9)** | **Framework C: *r*b** | **Frameworks** |
| --- | --- | --- | --- | --- |
| **Mean syllables per word** | **+0.188*** | **+0.833*** | **+0.414*** | **3/3** |
| Mean word length (characters) | +0.095** | +0.556* | +0.185** | **3/3** |
| Atmospheric word frequency (`sky_rate`) | −0.100** | −0.500(†) | −0.134* | **3/3** |

# **Appendix E. Summary of Indicators Reaching Significance in All Three Frameworks**

**Table E-1. All indicators reaching significance in all three analytical frameworks (3/3).**

| **#** | **Indicator** | **Hypothesis** | **Framework A: τ (*n* = 517)** | **Framework B: τ (*n* = 9)** | **Framework C: *r*b** | **Bootstrap 95% CI** | **Direction** |
| --- | --- | --- | --- | --- | --- | --- | --- |
| 1 | **Mean syllables per word** | C (*ānimitta*) | +0.188*** | +0.833*** | +0.414*** | [+0.059, +0.260] | ↑ |
| 2 | **NRC negative affect (composite)** | A (*ānxīn*) | −0.169*** | −0.722** | −0.255*** | [−0.240, −0.066] | ↓ |
| 3 | **Somatic word frequency** | B (*anātman*) | −0.158*** | −0.667* | −0.344*** | [−0.175, −0.052] | ↓ |
| 4 | **Sensory evenness** | C (*ānimitta*) | −0.119*** | −0.761** | −0.209*** | [−0.176, −0.036] | ↓ |
| 5 | Nature-to-self subject ratio | B (*anātman*) | −0.130*** | −0.556* | −0.168** | [−0.215, −0.049] | ↓ |
| 6 | **Tactile word frequency** | C (*ānimitta*) | −0.129*** | −0.761** | −0.235*** | [−0.178, −0.050] | ↓ |
| 7 | **Somatic-to-nature word ratio** | B (*anātman*) | −0.127*** | −0.611* | −0.283*** | [−0.141, −0.036] | ↓ |
| 8 | **Embodied sensory word frequency** | C (*ānimitta*) | −0.115*** | −0.778** | −0.192** | [−0.184, −0.027] | ↓ |
| 9 | **NRC sadness** | A (*ānxīn*) | −0.101** | −0.611* | −0.130* | [−0.155, −0.017] | ↓ |
| 10 | **Atmospheric word frequency** | C (*ānimitta*) | −0.100** | −0.500(†) | −0.134* | [−0.168, −0.011] | ↓ |
| 11 | **I/me/my pronoun frequency** | B (*anātman*) | −0.086** | −0.588* | −0.124* | [−0.150, +0.011] | ↓ |
| 12 | **Total sensory word frequency** | C (*ānimitta*) | −0.077* | −0.611* | −0.183** | [−0.140, +0.021] | ↓ |

*Note.* Ordered by |τ| (Framework A) in descending order. All indicators reach *p* < .05 (or meet the effect-size threshold) in all three independent analytical frameworks. Hypotheses: A = affective settling (*ānxīn*); B = self-effacement (*anātman*); C = perceptual transformation (*ānimitta*). NRC anger drops to 2/3 under the present periodization (Framework B remains significant; T1 vs. T3 is reduced to marginal significance) and is therefore not listed; I/me/my pronoun frequency, by contrast, is upgraded from 2/3 in the earlier scheme to 3/3 here.

# **Appendix F. Technical Details of the Statistical Methods**

**F.1. Data Preprocessing**

For indicators with strong right skew (skewness > 2.5), values were first Winsorized (truncated at the 1st and 99th percentiles) to limit the influence of extreme observations, then transformed using log(1 + *x*) to improve distributional shape. This preprocessing affects only the continuous-trend tests in Framework A; Frameworks B and C are based on ranks or medians and are unaffected by distributional skew.

**F.2. Framework A: Continuous-Trend Test**

Each indicator’s monotonic association with years of practice (composition year minus 1956) is tested using Kendall’s τ rank correlation. Kendall’s τ is based on the count of concordant and discordant observation pairs, requires no normality assumption, and assumes no linear functional form—making it appropriate for the non-normal distribution of word-frequency data in this study. Significance is evaluated at the two-tailed *p* < .05 level.

**F.3. Framework B: Collection-Level Trend**

The 517 poems are aggregated at the collection level (each indicator summarized by median or mean), and Kendall’s τ is computed over the nine collections ordered by year of publication (*n* = 9). This framework treats each collection as a single observation and is independent of Framework A.

**F.4. Framework C: Period Comparison**

The Mann-Whitney U test compares the distributions of T1 (236 poems) and T3 (136 poems), with the rank-biserial correlation (*r*b) reported as the effect-size measure. Values of *r*b range between −1 and +1; conventionally, |*r*b| < 0.1 is treated as a negligible effect, 0.1–0.3 as small, 0.3–0.5 as medium, and > 0.5 as large.

**F.5. Three-Period Omnibus Test**

Overall differences across T1, T2, and T3 are tested using the Kruskal-Wallis H test (a non-parametric analogue of one-way ANOVA), with *df* = 2. The KW statistics and *p*-values reported in the tables of Appendices B–D are derived from this test.

**F.6. Changepoint Detection**

The PELT (Pruned Exact Linear Time) algorithm (Killick et al., 2012) is applied to each core indicator. The procedure is as follows: the 517 poems are first binned by composition year using a 5-year window; the mean of each bin yields a 13-point aggregated time series. PELT is then run with the L2 (least-squares) cost function and a minimum segment length of two time points. A defining feature of this algorithm is that **the number of changepoints is not pre-specified by the analyst**: the penalty parameter automatically determines termination—when the residual reduction from adding a changepoint no longer offsets the penalty, the algorithm stops.

The detection results (Table F-1) show that at pen = 10—the value used for the bootstrap analysis—three indicators independently identify two changepoints: NRC negative-emotion frequency, I/me/my pronoun frequency, and somatic word frequency, with locations in the 1970–1975 and 1995–2000 between-bin intervals (5-year binning resolution). Sensitivity is not identical across all penalties: the pronoun and somatic series retain both locations from pen = 1 through pen = 20, whereas at pen = 20 the negative-emotion series retains only the earlier changepoint (1970). Mean syllables per word and the nature-to-self subject ratio yield no changepoints at any tested penalty. The earlier location falls close to the T1/T2 boundary derived from biographical evidence, while the later one falls more than a decade after the T2/T3 boundary; the changepoint evidence is accordingly treated as corroborating the first boundary only (see §5.4).

**Table F-1. PELT changepoint detection results (L2 cost function, min_size = 2).**

| **Indicator** | **pen = 1** | **pen = 5** | **pen = 10** | **pen = 20** |
| --- | --- | --- | --- | --- |
| NRC negative-emotion frequency | 1970, 1995 | 1970, 1995 | 1970, 1995 | 1970 |
| I/me/my pronoun frequency | 1970, 1995 | 1970, 1995 | 1970, 1995 | 1970, 1995 |
| Somatic word frequency | 1970, 1995 | 1970, 1995 | 1970, 1995 | 1970, 1995 |
| Mean syllables per word | — | — | — | — |
| Nature-to-self subject ratio | — | — | — | — |

**Penalty parameter selection. PELT’s `pen` parameter governs the penalty applied to each additional changepoint: larger values yield more conservative detection (fewer changepoints). Table F-1 spans pen = 1 to pen = 20, covering the range from lenient to strict sensitivity. The pronoun and somatic series retain the 1970 and 1995 locations at every tested value; the negative-emotion series retains both locations at pen = 1, 5, and 10 but only the earlier location at pen = 20. The earlier location is therefore robust across all three detected indicators and all tested penalties, whereas the later negative-emotion changepoint is less robust under the strictest penalty.**

**Bootstrap confidence intervals.** To assess statistical uncertainty in the changepoint locations, 1,000 bootstrap resamples were drawn (with replacement) from the 517 poems; for each resample, the time series was rebinned and PELT (pen = 10) re-run. The resulting distribution of changepoint locations is reported in Table F-1b.

**Table F-1b. Bootstrap analysis of changepoint locations (1,000 resamples, pen = 10).**

| **Indicator** | **Changepoint** | **Median** | **Modal location (%)** | **95% CI** |
| --- | --- | --- | --- | --- |
| NRC negative-emotion frequency | 1st | 1970 | 1970 (87%) | [1970, 1995] |
|  | 2nd | 1995 | 1995 (76%) | [1995, 2000] |
| I/me/my pronoun frequency | 1st | 1970 | 1970 (98%) | [1970, 1970] |
|  | 2nd | 1995 | 1995 (93%) | [1995, 2000] |
| Somatic word frequency | 1st | 1970 | 1970 (99%) | [1970, 1970] |
|  | 2nd | 1995 | 1995 (87%) | [1995, 2000] |

The first changepoint is highly stable across all three indicators, with 87–99% of bootstrap samples localizing it to 1970. The second changepoint is stable within the 1995–2000 range. These changepoints are independently identified by the algorithm rather than imposed a priori; the procedure is independent of the three-period scheme and serves to establish where the statistically identified turning points fall relative to the biographically derived periodization, which they do for the first boundary but not for the second. Implementation uses the Python `ruptures` library (Truong et al., 2020).

**F.7. Residual Autocorrelation Function Diagnostics (Figure F-1)**

To assess whether time-series autocorrelation in Framework A might bias the significance tests for Kendall’s τ, the residual autocorrelation function (ACF) was computed for each of the 12 indicators that reached significance in all three frameworks. The procedure was as follows: a monotonic trend was fitted with years of practice as the predictor; the residual series was extracted; and autocorrelation coefficients for lag 1 through lag 8 were computed together with the 95% confidence band based on the Bartlett formula.

Across all 12 indicators, autocorrelation coefficients at lag > 1 remain within ±0.12, with only four of the 84 coefficients at these lags touching or slightly exceeding the Bartlett 95% band, scattered across different indicators and lags; lag 1 shows at most mild autocorrelation (|r| < 0.15). This pattern indicates the absence of a sustained AR structure, so that standard Kendall’s τ significance estimates are not materially inflated. Combined with the collection-blocked bootstrap CIs reported in §F.12, this confirms that the core findings are not driven by time-series dependence.

*Figure F-1. Residual autocorrelation function (lag 1–8) for the 12 core indicators, with Bartlett 95% bands.*

Note on Figure F-1. Residuals are taken from a least-squares linear detrend on years of practice (a linear trend being monotonic by construction), with poems ordered by composition year; dashed lines give the Bartlett 95% band. The figure and the underlying coefficients are produced by scripts/phase_robust_acf.py in the Supplementary Material, which reads only the per-poem feature tables in results/ and therefore runs without the poem texts; the per-indicator values are written to results/residual_acf.csv.

**F.8. Syntactic-Subject Analysis Algorithm**

Each poem is sentence-tokenized (sent_tokenize) and POS-tagged (pos_tag) using the Python NLTK toolkit. For each sentence, the first six tokens are inspected, and the first pronoun (PRP/PRP$) or noun (NN/NNS/NNP/NNPS) encountered is taken as the grammatical subject. Subjects are classified into four categories using predefined wordlists: self-subject (I/me/my, etc.), human-subject (he/she/they, etc.), nature-subject (mountain/wind/river, etc.; 100+ entries), and impersonal-subject (it/there, etc.). Sentences whose subject does not match any category are excluded from the statistical analysis.

**F.9. Multiple-Comparisons Correction**

Framework A (Kendall’s τ continuous-trend test) involves a total of 25 hypothesis tests across this study. To control false-positive inflation under multiple comparisons, the Benjamini-Hochberg FDR procedure (Benjamini & Hochberg, 1995) was applied to all 25 *p*-values, controlling the false discovery rate at α = .05.

The correction results (Table F-2): of the 25 tests, 17 are significant before correction (*p* < .05), and 15 remain significant after correction. Only NRC surprise (*p*_raw = .049 → *p*_adj = .071) and tentativeness word frequency (*p*_raw = .046 → *p*_adj = .071)—both marginal—lose significance. **All 12 indicators that reached cross-framework consistency (3/3) remain significant after FDR correction**, indicating that the core findings are robust to multiple-comparisons concerns.

**Table F-2. Benjamini-Hochberg FDR correction (Framework A Kendall’s τ, *n* = 517; 25 tests).**

| **Indicator** | **τ** | ***p*_raw** | **sig** | ***p*_adj** | **sig_adj** |
| --- | --- | --- | --- | --- | --- |
| Mean syllables per word | +0.188 | <.001 |  | <.001 |  |
| NRC negative affect (composite) | −0.169 | <.001 |  | <.001 |  |
| Somatic word frequency | −0.158 | <.001 |  | <.001 |  |
| Tactile word frequency | −0.129 | <.001 |  | <.001 |  |
| Nature-to-self subject ratio | −0.130 | <.001 |  | .001 |  |
| Somatic-to-nature word ratio | −0.127 | <.001 |  | <.001 |  |
| Sensory evenness | −0.119 | <.001 |  | .001 |  |
| NRC disgust | −0.113 | <.001 |  | .001 |  |
| NRC sadness | −0.101 | .001 |  | .003 |  |
| Atmospheric word frequency | −0.100 | .001 |  | .004 |  |
| NRC anger | −0.094 | .003 |  | .006 |  |
| NRC fear | −0.088 | .004 |  | .009 |  |
| I/me/my pronoun frequency | −0.086 | .007 |  | .014 |  |
| Certainty-to-tentativeness ratio | −0.085 | .008 |  | .014 |  |
| Total sensory word frequency | −0.077 | .011 |  | .019 |  |
| NRC surprise | −0.061 | .049 |  | .071 | n.s. |
| Tentativeness word frequency | +0.065 | .046 |  | .071 | n.s. |
| Certainty word frequency | −0.049 | .126 | n.s. | .162 | n.s. |
| NRC joy | −0.046 | .129 | n.s. | .162 | n.s. |
| Visual word frequency | −0.047 | .130 | n.s. | .162 | n.s. |
| Olfactory word frequency | −0.034 | .327 | n.s. | .389 | n.s. |
| Auditory word frequency | −0.025 | .454 | n.s. | .516 | n.s. |
| NRC trust | +0.021 | .493 | n.s. | .536 | n.s. |
| NRC positive (composite) | −0.001 | .978 | n.s. | .989 | n.s. |
| NRC anticipation | +0.000 | .989 | n.s. | .989 | n.s. |

*Note.* *p < .05, **p < .01, ***p < .001. `sig`: uncorrected significance; `sig_adj`: BH-FDR–corrected significance. Ordered by |τ| descending.

**F.10. Inter-Rater Agreement and Disagreement Resolution (Cohen’s κ)**

Inter-rater agreement was assessed for the three domain-specific lexicons. After the first researcher constructed the initial lexicons, the second researcher independently rendered binary judgments (include/exclude) on each entry. To ensure that the κ statistic could be computed, 20 neutral control words (judged “exclude” by both raters) were appended to each lexicon; control words included: *thought, word, memory, meaning, always, never, perhaps, though, while, before, after, until, because, without, beyond, itself, become, remain, already, toward*.

**Table F-3. Inter-rater agreement for the three domain-specific lexicons.**

| **Lexicon** | **Total entries (incl. controls)** | **Agreements** | **Disagreements** | **Cohen’s κ** | **Percent agreement** |
| --- | --- | --- | --- | --- | --- |
| Body lexicon | 82 (62 + 20) | 82 | 0 | 1.000 | 100.0% |
| Nature lexicon | 204 (184 + 20) | 199 | 5 | 0.875 | 97.5% |
| Sensory lexicon | 53 (33 + 20) | 50 | 3 | 0.883 | 94.3% |
| **Overall** | **339** | — | **8** | **0.923** | **97.6%** |

*Note.* κ ≥ 0.80 indicates “excellent” reliability (Landis & Koch, 1977). The body lexicon showed perfect agreement between the two raters (κ = 1.00); the nature and sensory lexicons each had a small number of disagreements, all resolved by consensus and retained.

**Table F-3b. The eight disagreement entries.**

| **Entry** | **Lexicon** | **Rater 1** | **Rater 2** | **Resolution** |
| --- | --- | --- | --- | --- |
| *bright* | sensory | include | exclude | Include (visual intensity, a typical sensory experience word) |
| *dark* | sensory | include | exclude | Include (visual intensity, a typical sensory experience word) |
| *light* | sensory | include | exclude | Include (visual intensity, a typical sensory experience word) |
| *bay* | nature | include | exclude | Include (a nature-topographical term: inlet/harbor) |
| *brush* | nature | include | exclude | Include (vegetation/thicket; the dominant usage) |
| *fly* | nature | include | exclude | Include (bird/insect; the dominant usage in Snyder’s poetry) |
| *sage* | nature | include | exclude | Include (herb/sagebrush; an ecologically representative plant of the American West) |
| *spring* | nature | include | exclude | Include (water source/season; the dominant usage in nature-related contexts) |

*Note.* Rater 2 treated *bright/dark/light* as general intensity adjectives rather than sensory-exclusive terms, and treated *bay/brush/fly/sage/spring* as polysemous (e.g., *fly* as a verb of motion, *spring* as a verb meaning to leap). After discussion, all entries were retained in their respective lexicons in light of contextual usage in the Snyder corpus.

**F.11. Lexicon Validity: Comparison with the Empath Open-Source Tool**

To independently assess the directional validity of the domain-specific lexicons, each lexicon’s years-of-practice trend (Framework A Kendall’s τ, *n* = 517) was compared with the closest available category in Empath (Fast et al., 2016), an open-source lexical analysis tool with 194 predefined categories. Empath does not include a “perception” category, so the sensory composite was constructed as the sum of the *cold*, *warmth*, *pain*, and *smell* category scores. Four indicator pairs were compared, covering the body, sensory, negative-emotion, and sadness dimensions. Collection-level associations were computed using Spearman’s *r* (*n* = 9 collections, ordered by publication year).

**Table F-4. Comparison with Empath (poem-level *n* = 517; collection-level *n* = 9).**

| **Indicator pair** | **Domain-specific τ** | **Empath τ** | **Direction agrees** | **Collection-level Spearman *r*** | ***p*** |
| --- | --- | --- | --- | --- | --- |
| Somatic ↔ Empath body | −0.158 | −0.145 | ✓ | +0.525 | .147 |
| Embodied sensory ↔ Empath sensory composite† | −0.116 | −0.149 | ✓ | +0.803** | .009 |
| NRC negative ↔ Empath negative_emotion | −0.169 | −0.147 | ✓ | +0.730* | .025 |
| NRC sadness ↔ Empath sadness | −0.101 | −0.060 | ✓ | — | — |

*Note.* †Sensory composite = Empath cold + warmth + pain + smell. The fourth pair (NRC sadness ↔ Empath sadness) cannot be computed at the collection level because Empath sadness has insufficient variance at that level: the category registers in only 8.7% of poems, so all nine collection-level medians are zero. Directional agreement: 4/4 (100%); collection-level *r* range: +0.525–+0.803 (2 of 3 computable pairs reach *p* < .05). *p < .05; **p < .01.

**F.12. Collection-Blocked Bootstrap Confidence Intervals**

To assess the influence of within-collection clustering on the Framework A Kendall’s τ estimates, a block bootstrap was implemented for all 12 indicators that reached significance in all three frameworks. The nine collections served as resampling units: 1,000 bootstrap samples were drawn with replacement (each sample drawing nine collections, with replacement allowed); within each sample, all poems from the drawn collections were pooled and Kendall’s τ was recomputed (with years of practice as the continuous predictor); the 2.5th and 97.5th percentiles of the bootstrap distribution were taken as the 95% CI (percentile method). This procedure controls for within-collection clustering and tests the robustness of monotonic-trend estimates under collection-level random variation.

**Table F-5. Block bootstrap 95% CIs for the 12 core indicators (collection-blocked, 1,000 resamples, percentile method).**

| **#** | **Indicator** | **Hypothesis** | **Framework A τ** | **Bootstrap 95% CI** | **CI spans zero** |
| --- | --- | --- | --- | --- | --- |
| 1 | Mean syllables per word | C (*ānimitta*) | +0.188 | [+0.059, +0.260] | No |
| 2 | NRC negative affect (composite) | A (*ānxīn*) | −0.169 | [−0.240, −0.066] | No |
| 3 | Somatic word frequency | B (*anātman*) | −0.158 | [−0.175, −0.052] | No |
| 4 | Sensory evenness | C (*ānimitta*) | −0.119 | [−0.176, −0.036] | No |
| 5 | Nature-to-self subject ratio | B (*anātman*) | −0.130 | [−0.215, −0.049] | No |
| 6 | Tactile word frequency | C (*ānimitta*) | −0.129 | [−0.178, −0.050] | No |
| 7 | Somatic-to-nature word ratio | B (*anātman*) | −0.127 | [−0.141, −0.036] | No |
| 8 | Embodied sensory word frequency | C (*ānimitta*) | −0.115 | [−0.184, −0.027] | No |
| 9 | NRC sadness | A (*ānxīn*) | −0.101 | [−0.155, −0.017] | No |
| 10 | Atmospheric word frequency | C (*ānimitta*) | −0.100 | [−0.168, −0.011] | No |
| 11 | I/me/my pronoun frequency | B (*anātman*) | −0.086 | [−0.150, +0.011] | Yes |
| 12 | Total sensory word frequency | C (*ānimitta*) | −0.077 | [−0.140, +0.021] | Yes |

*Note.* Of the 12 indicators, 10 have bootstrap 95% CIs that exclude zero, confirming that effect sizes remain robust after controlling for collection-level clustering. The remaining two (I/me/my pronoun frequency and total sensory word frequency) have CIs that span zero, reflecting greater uncertainty for these weaker effects under collection-blocked resampling; for both, however, the point-estimate direction is consistent across all three frameworks, and they are characterized as “moderate evidence” rather than “robust evidence.”

# **Appendix G. Comparison Case (Wendell Berry)**

To probe whether the affective and self-referential trends are specific to contemplative practice rather than to aging or to the nature-poetry genre, the same indicators and statistical procedures were applied to Wendell Berry (a contemporaneous, non-practising nature poet; 280 poems, 1964–2010). Table G-1 reports the two cross-poet frameworks for the core indicators (see also Table 2 and §5.5).

**Table G-1. Comparison case (Wendell Berry) vs. Snyder: two-framework results for the core indicators.**

| **Indicator** | **Snyder τ (A)** | **Snyder r_b (C)** | **Berry τ (A)** | **Berry r_b (C)** | Δτ (A) [95% CI] | **Interpretation** |
| --- | --- | --- | --- | --- | --- | --- |
| NRC negative affect | -0.169*** | -0.255*** | -0.055 n.s. | -0.106 n.s. | −0.113 [−0.212, +0.006] | Practice-consistent (descriptive) |
| First-person pronouns (I/me/my) | -0.086** | -0.126* | +0.001 n.s. | -0.077 n.s. | −0.087 [−0.245, +0.049] | Practice-consistent (descriptive) |
| Certainty / tentativeness ratio | -0.085** | -0.181** | -0.065 n.s. | -0.156* | −0.020 [−0.165, +0.184] | Practice-consistent (descriptive) |
| Mean syllables per word | +0.188*** | +0.414*** | +0.054 n.s. | +0.156 n.s. | +0.134 [−0.007, +0.251] | Practice-consistent (descriptive) |
| Somatic (body) words | -0.158*** | -0.344*** | -0.210*** | -0.317*** | +0.052 [−0.076, +0.228] | Shared (descriptive) |
| Atmospheric words | -0.100** | -0.130* | -0.205*** | -0.339*** | +0.105 [−0.047, +0.250] | Shared (descriptive) |
| Total sensory words | -0.077* | -0.183** | -0.170*** | -0.303*** | +0.093 [−0.078, +0.258] | Shared (descriptive) |
| Tactile words | -0.129*** | -0.235*** | -0.121** | -0.192* | −0.008 [−0.136, +0.128] | Shared (descriptive) |
| Nature-to-self subject ratio | -0.130*** | -0.167** | -0.180*** | -0.274*** | +0.049 [−0.119, +0.193] | Shared (descriptive) |

Note. Framework A = Kendall's τ over career time (continuous; Snyder n = 517, Berry n = 280); Framework C = rank-biserial r from a Mann–Whitney U comparison of the early (T1) and late (T3) periods. Collection-level results (Framework B) for Snyder appear in Appendices B–E. "Practice-consistent (descriptive)" labels a within-poet pattern—significant trend in Snyder, none in Berry—and carries no inferential claim about the between-poet difference; "Shared (descriptive)" labels significant within-poet trends in both poets. Δτ = τ(Snyder) − τ(Berry) (Framework A), with percentile 95% CIs from a collection-blocked bootstrap (B = 20,000; collections resampled with replacement independently within each poet; script snyder_berry_difference_test.py, results snyder_berry_difference_test.csv in the supplementary package); the percentile construction is the pre-specified primary analysis. Sensitivity intervals from the same resampling scheme behave as follows: basic (reflected-percentile) intervals exclude zero for negative-emotion frequency ([−0.233, −0.015]) and mean syllables per word ([+0.016, +0.274]); standard multi-sample BCa intervals (scipy.stats.bootstrap) exclude zero for negative-emotion frequency ([−0.236, −0.016]) but include it for mean syllables per word ([−0.002, +0.257]), whose BCa lower endpoint is numerically indistinguishable from zero across Monte Carlo seeds. Conclusions for these two indicators are therefore sensitive to interval construction and are treated as suggestive rather than established; for the remaining seven indicators all three constructions agree in including zero. All comparisons are additionally subject to the temporal-index asymmetry described in §4.5 (Berry: volume publication years). *p < .05, **p < .01, ***p < .001.

**Data and Code Availability.** All analytical scripts (Python) and intermediate results (.xlsx) used in this study are archived in the project directory and available for inspection and replication. Core scripts include: phase2_word_freq.py (word-frequency analysis), phase_nrc.py (NRC emotion analysis), phase_spacy.py (syntactic-subject analysis), phase_supplement.py (five-sense and epistemic-stance analysis), phase_auto2.py (nature vocabulary and syllable analysis), phase_robust.py (three-framework robustness checks), and supplementary_analyses.py (lexicon reliability and validity, bootstrap CIs, Empath comparison). NRC Emotion Lexicon: Mohammad and Turney (2013); Empath: Fast et al. (2016).
